# Effect of Short Fiber Reinforcements on Fracture Performance of Cement-Based Materials: A Systematic Review Approach

**DOI:** 10.3390/ma14071745

**Published:** 2021-04-01

**Authors:** Waqas Ahmad, Mehran Khan, Piotr Smarzewski

**Affiliations:** 1 Department of Civil Engineering, COMSATS University Islamabad, Abbottabad 22060, Pakistan; waqasahmad@cuiatd.edu.pk; 2 Department of Civil Engineering, Dalian University of Technology, Dalian 116024, China; 3 Department of Structural Engineering, Faculty of Civil Engineering and Architecture, Lublin University of Technology, 20-618 Lublin, Poland

**Keywords:** fracture characteristics, cement-based material, fibers, fiber-reinforced cementitious composites, fracture properties

## Abstract

Fracture characteristics were used to effectively evaluate the performance of fiber-reinforced cementitious composites. The fracture parameters provided the basis for crack stability analysis, service performance, safety evaluation, and protection. Much research has been carried out in the proposed study field over the previous two decades. Therefore, it was required to analyze the research trend from the available bibliometric data. In this study, the scientometric analysis and science mapping techniques were performed along with a comprehensive discussion to identify the relevant publication field, highly used keywords, most active authors, most cited articles, and regions with largest impact on the field of fracture properties of cement-based materials (CBMs). Furthermore, the characteristic of various fibers such as steel, polymeric, inorganic, and carbon fibers are discussed, and the factors affecting the fracture properties of fiber-reinforced CBMs (FRCBMs) are reviewed. In addition, future gaps are identified. The graphical representation based on the scientometric review could be helpful for research scholars from different countries in developing research cooperation, creating joint ventures, and exchanging innovative technologies and ideas.

## 1. Introduction

In civil engineering applications, cement-based materials (CBMs) are substantially used due to their low cost, simple production, and extensive sources [1,2,3,4,5,6,7,8]. However, the factors limiting their further applications include their brittle nature, low tensile strength, weak resistance to cracks, low energy absorption, and small strain capacity [9,10,11,12]. Fibers are incorporated into CBMs as reinforcement to improve their mechanical properties [13,14,15,16,17,18,19,20,21,22,23]. Fibers provide a bridging effect in the matrix to resist crack propagation and distribute stresses [24,25,26]. Nowadays, natural and artificial fibers are used in fiber-reinforced cement-based materials (FRCBMs), particularly to enhance their fracture properties [27,28,29,30,31,32,33,34]. Among the artificial fibers, steel fibers have been widely, practically use in members (both structural and non-structural) to improve their properties, including resistance to crack propagation, toughness, and impact resistance [35,36]. Steel fibers have good stability, ample interaction with matrixes, and better mechanical properties [5,37]. In addition to steel fibers, various other fiber types are used in FRCBMs [38,39,40,41,42]. Furthermore, incorporating micro or nano fibers as reinforcement into CBMs has gotten more attention due to their additional functions, e.g., electric conductivity and crack assessment capability. Generally, using a single fiber can provide reinforcement up to some scale/level, and it is difficult to provide crack resistance at other scales/levels [43,44]. The cracking of CBMs is a multi-scale and continuous failure process due to their multi-stage behavior [11,45,46,47]. Therefore, fiber hybridization, i.e., the use of distinct fiber types in CBMs, has been applied to enhance both strength and toughness at once [48,49]. Fiber hybridization can be of three types: (i) different types of fibers with the same scale or length [50], (ii) different fiber types with dissimilar scales or lengths [33,51,52], and (iii) the same fiber type with dissimilar scales or lengths [28,53]. The incorporation of different types of fibers in CBMs can reveal different positive synergy effects [43].

A CBM is a polyphase composite with inner cracks and inborn imperfections/defects [54]. Fracture mechanics is a highly effective method to examine the performance of a material in a structure. Fracture mechanics provides the basis for crack stability analysis, service performance, safety evaluation, and protection [54,55]. An FRCBM is commonly used in civil engineering structures such as bridge girders, tunnel lining, runways, and anti-explosive structures because it exhibits excellent mechanical and durability properties [4,7]. Due to the extensive applications of FRCBMs in the construction industry, particularly with the advancement of high-performance concrete (HPC) and ultra-high-performance concrete (UHPC), extra focus has been given to the safety evaluation of FRCBMs. The polyphase behavior of FRCBMs has a substantial effect on congenital and arbitrary imperfections that may lead to the fracture breakdown of FRCBMs. For the evaluation of structural safety and crack analysis, research has focused on the investigation of fracture properties of FRCBMs using fracture mechanics [7,9,11,24,50,52,53,54].

To create a profound and clear link between different aspects of the existing literature, manual reviews are not sufficient. Nowadays, scientific mapping and network visualization between bibliographic coupling, co-citations, and co-occurrence are the demanding aspects of modern research. The scientometric review can deal with huge amounts of data without making further complications to provide answers to the fundamental limitations of former manual reviews. In this study, in addition to the conventional review, scientometric analysis was also conducted to give a solution to the fundamental limitations of conventional reviews. More clearly, the author’s synergy, article co-citations, keyword co-occurrence, and visualization of active countries researching in the field of the fracture properties of FRCBMs were thoroughly analyzed. The scientometric analysis was performed along with comprehensive discussions for the present study to achieve the following objectives:

◦ To identify the relevant publication field, highly used keywords, most active authors, most cited articles, and regions with the largest impact on the field of the fracture properties of FRCBMs.
◦ To observe the present research state and its focus on various factors during the last two decades.
◦ To find gaps in the existing research to guide the directions for future research.

To date, a huge number of publications are available on the fracture properties of FRCBMs containing various fibers to investigate their fracture properties. However, most of the studies carried out so far have been on the fracture properties of plain concrete (PC). This review gives information on the fracture properties of FRCBMs. The graphical representation based on a scientometric review may aid researchers from various countries in establishing research collaborations, forming joint ventures, and sharing innovative technologies and ideas. Furthermore, the factors influencing the fracture properties of FRCBMs are reported, and strengthening technique for enhancing the fracture properties of FRCBMs are discussed. Finally, recommendations are given for the upcoming studies. The major aim of the current study was to deliver an overview of the present approach over the fracture properties of FRCBMs and factors affecting the fracture behavior of FRCBMs. This review provides information that will contribute to the understanding, evaluation, and controlling of FRCBM fracture properties, as well as providing advantages to the field of CBM structures.

## 2. Experimental Strategies

In this study, two approaches were adopted: a scientometric-based review [56,57,58,59] and comprehensive discussion on the influencing factors of FRCBM fracture properties. The main reason for adopting the scientometric review was that several review-based studies in civil engineering have revealed that in manual analysis, researchers may depend upon judgments that may be subjective and, therefore, unreliable. A scientometric analysis can provide an impartial and less subjective result [60,61,62]. This approach was suitable for the current study because it analyzed and highlighted the research growth over a period of two decades. With this method, the quantitative analysis of research visualized maps and linked the research development from a large amount of bibliometric data to evaluate the research growth.

**Figure 1:**
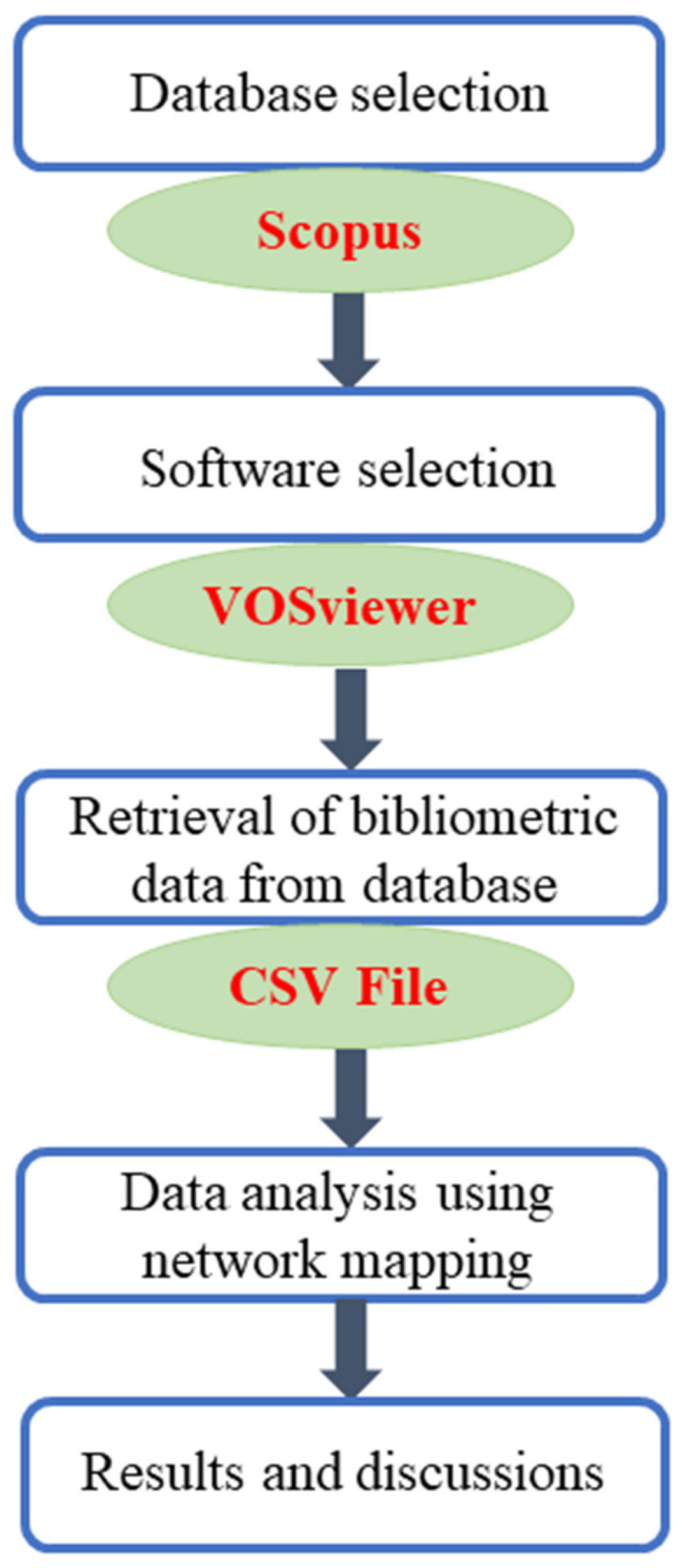
Scientometric analysis sequence. CSV: comma separated values.

Researchers have published a huge number of articles, and it is essential to identify the most reliable database. For a literature search, the two most efficient, extensive, and objective databases, as suggested by Aghaei et al. [63], are Web of Science and Scopus. Scopus has a wider coverage and more updated bibliometric data than Web of Science [63,64,65]. In the present study, Scopus was deployed to extract the bibliometric data on the fracture properties of FRCBMs. To exclude the irrelevant publications, data-refining options were checked out. In the “document type,” only articles and review were selected. The “source type” was set to journal only while keeping the “language” as English. For the extraction of relevant articles from the Scopus database, the searched keywords included “concrete fracture properties,” “cementitious composite fracture properties,” “cement-based material fracture properties,” “fiber-reinforced concrete fracture,” and “hybrid fiber-reinforced concrete fracture.” In past studies, similar approaches were already adopted by researchers in various fields [66,67]. A scientometric review adopt science mapping, which is used by researchers for the analysis of bibliometric data for various purposes [68] and which reports the challenges faced by researchers while creating a relationship between keywords, authorship, and countries with manual reviews in specified research fields [69]. The relevant data from the Scopus were saved in comma separated values (CSV) format for further analysis using suitable software. The software used for the generation of science mapping and visualization was VOSviewer (version 1.6.16, Leiden University, Leiden, The Netherlands). VOSviewer is an open-source tool, which is highly recommended in the literature, that has versatile features for mapping and has been used widely in various fields [70,71,72,73,74]. Therefore, to achieve the objectives of the current study, the VOSviewer was used. The CSV file was imported to VOSviewer and analyzed in a few steps while ensuring consistency and reliability in data. The science mapping analysis was performed to determine keyword co-occurrence, citation network, co-authorship, documents, bibliometric overlapping, and country citations. The numbers of citations were also recorded. Furthermore, links between authors, publications, and countries were detailed. Maps were generated to visualize all the parameters, their links, and co-occurrence, while their corresponding quantitative values were summarized in tables. To develop the key research themes, the keywords were also analyzed and are thoroughly outlined in discussion section. The sequence of the scientometric analysis is shown in Figure 1.

## 3. Science Mapping Results and Discussions

### 3.1. Publication Area and Annual Trend

**Figure 2:**
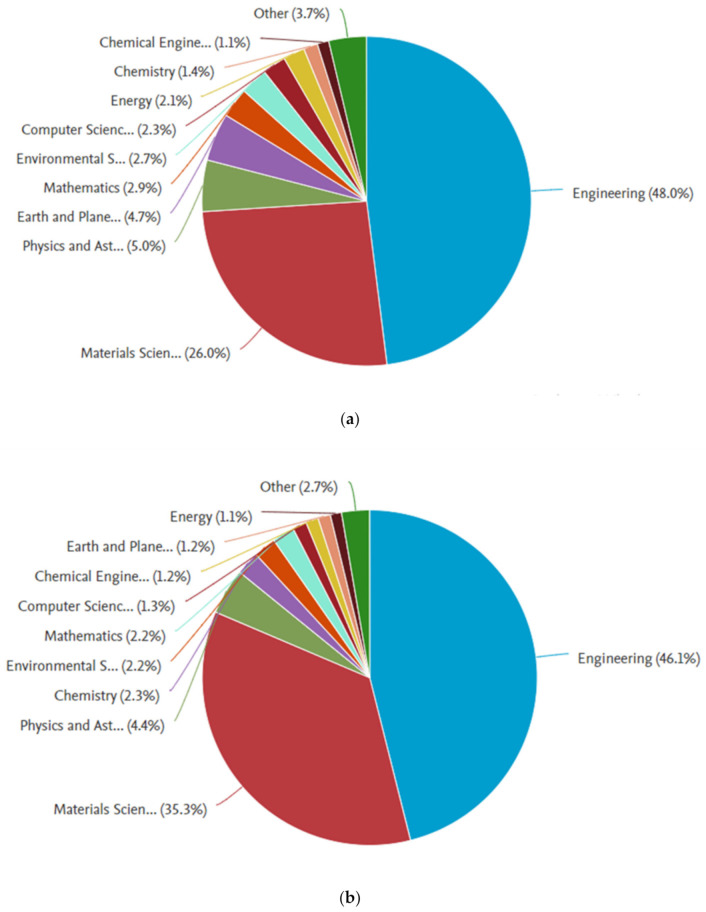

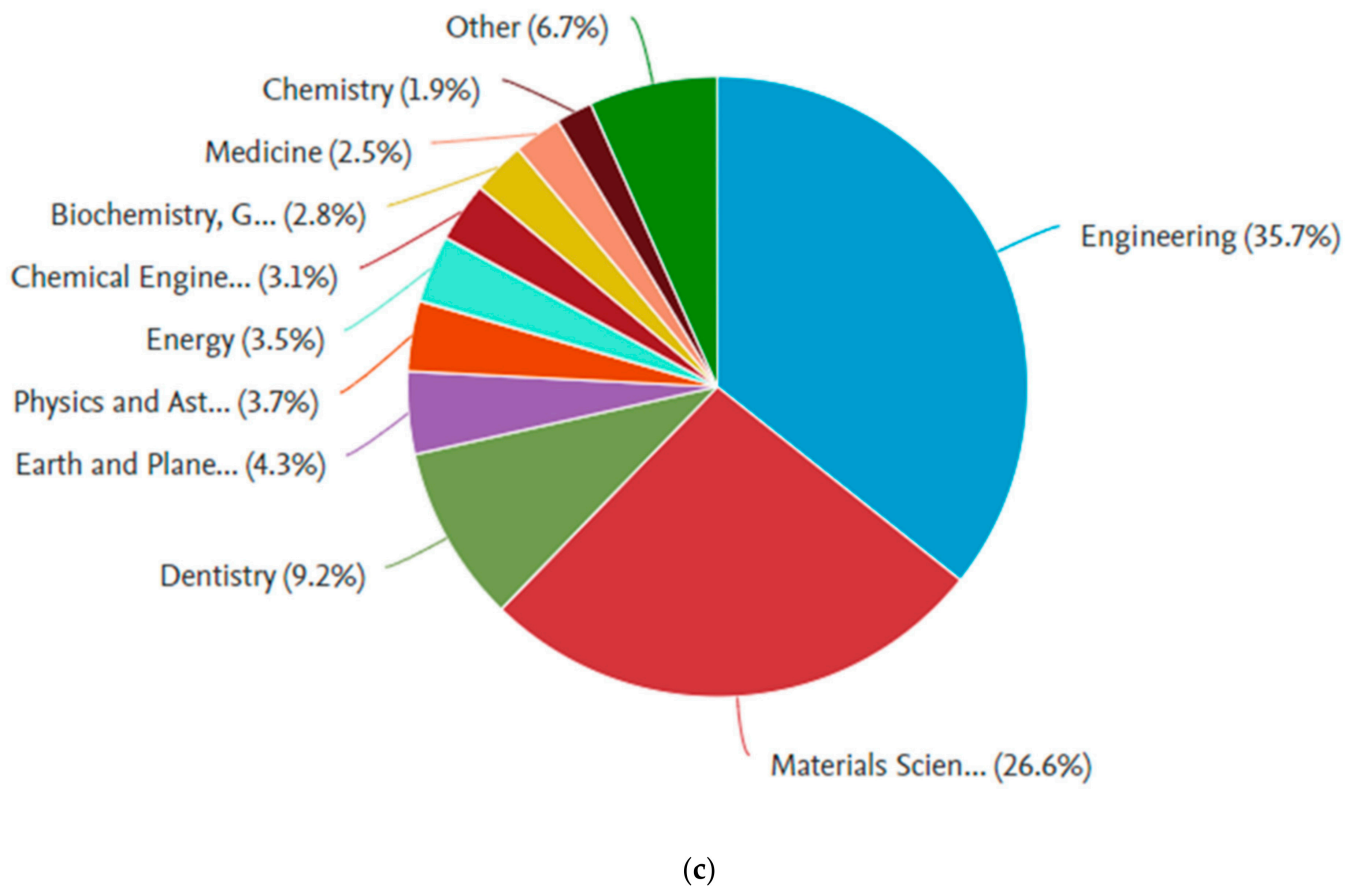
Subject distribution of publications on fracture properties: (**a**) concrete, (**b**) cementitious composites, and (**c**) cement-based material.

**Figure 3:**
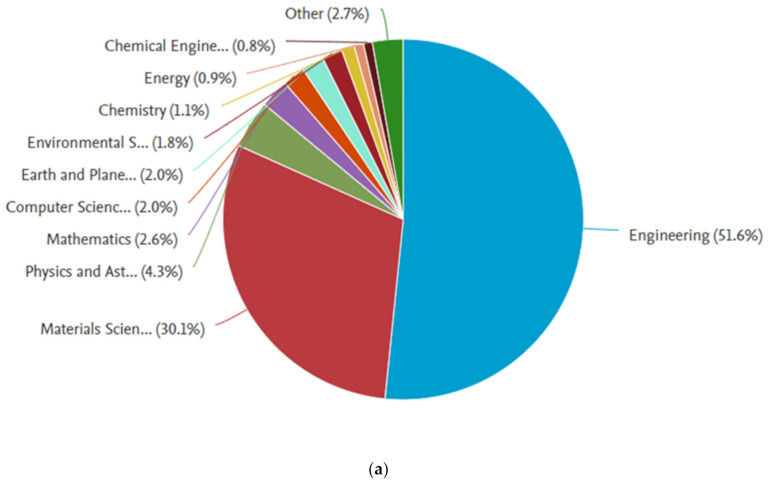

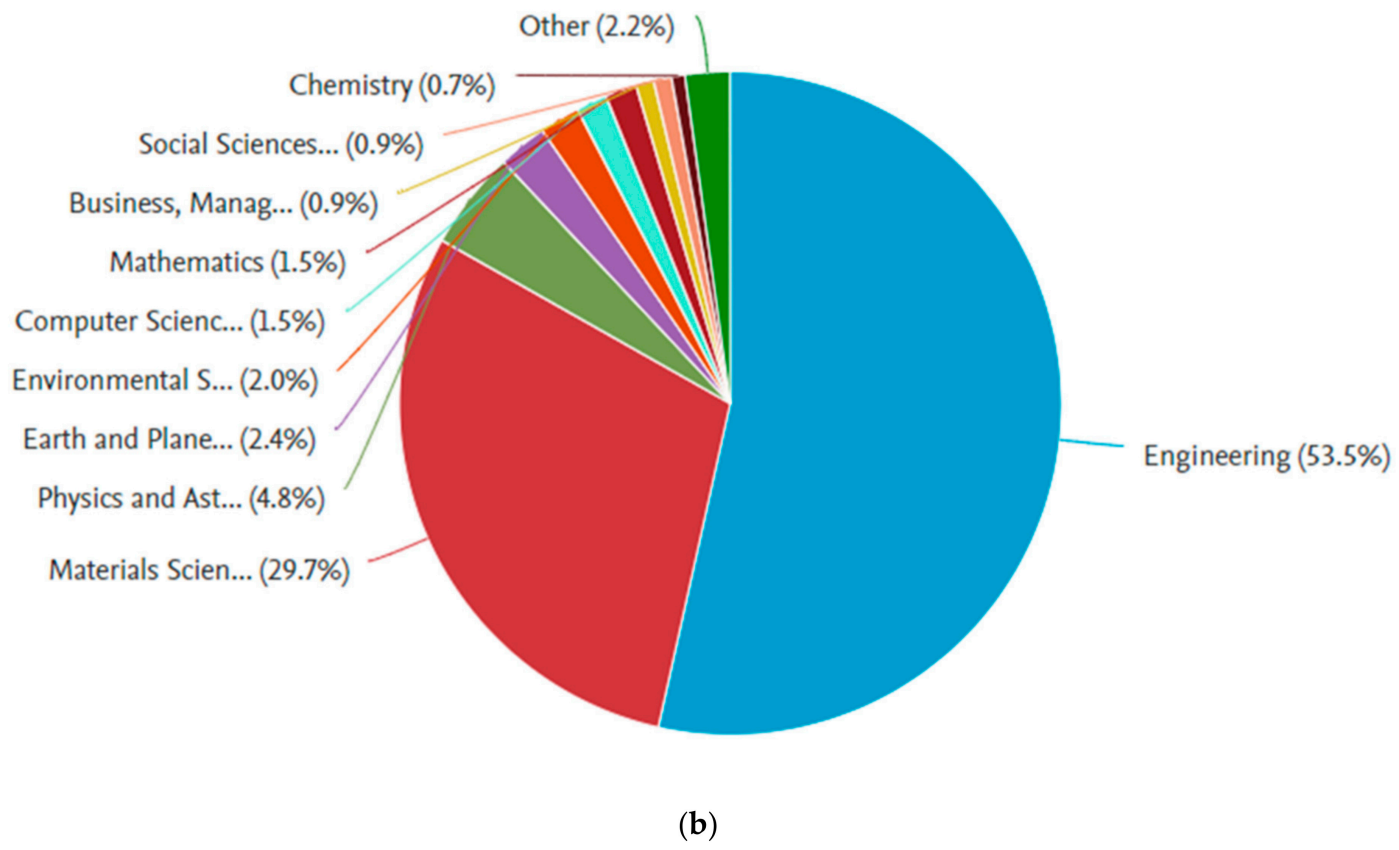
Subject distribution of publications on fracture properties: (**a**) fiber-reinforced concrete and (**b**) hybrid fiber-reinforced concrete.

**Figure 4:**
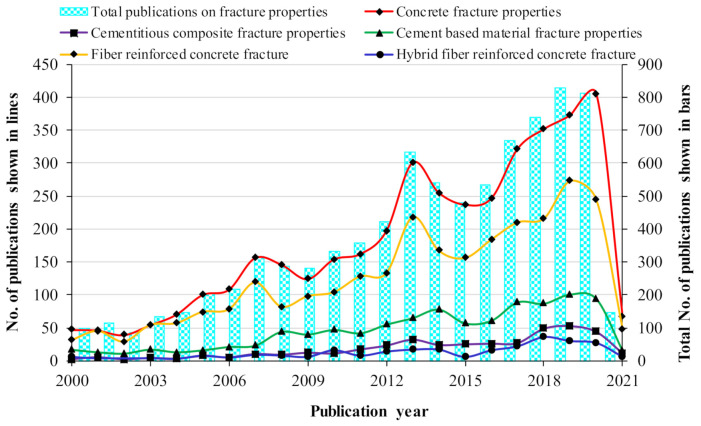
Annually published articles.

The data collected from the Scopus database were analyzed by the Scopus analyzer to know the most relevant research areas. From the analysis, it was revealed that the relevant publications have mostly been in the Engineering and Materials Science field. The Engineering and Materials Science field was found to contain around 74.0%, 81.4%, and 62.3% of the publications related to the fracture properties of concrete, cementitious composites, and cement-based materials, respectively, as shown in Figure 2. Moreover, this field was found to contain around 81.7% and 83.2% of publications related to the fracture properties of fiber-reinforced concrete and hybrid fiber-reinforced concrete, respectively, as shown in Figure 3. It is important to mention that the time duration limit was applied for the retrieval of bibliometric data, i.e., to start from 2000. The annual publication trend in the current study field from 2000 to 2021 (January) is shown in Figure 4. A gradual increase in publications in the field of engineering and material science on the fracture properties of CBMs and FRCBMs could be observed. However, an abrupt hike was observed in the last decade. It is fascinating to know that researchers have been analyzing these fracture properties for the safety evaluation and assessment of structural and non-structural members’ service performance.

### 3.2. Keyword Co-Occurrence Scientific Mapping

**Figure 5:**
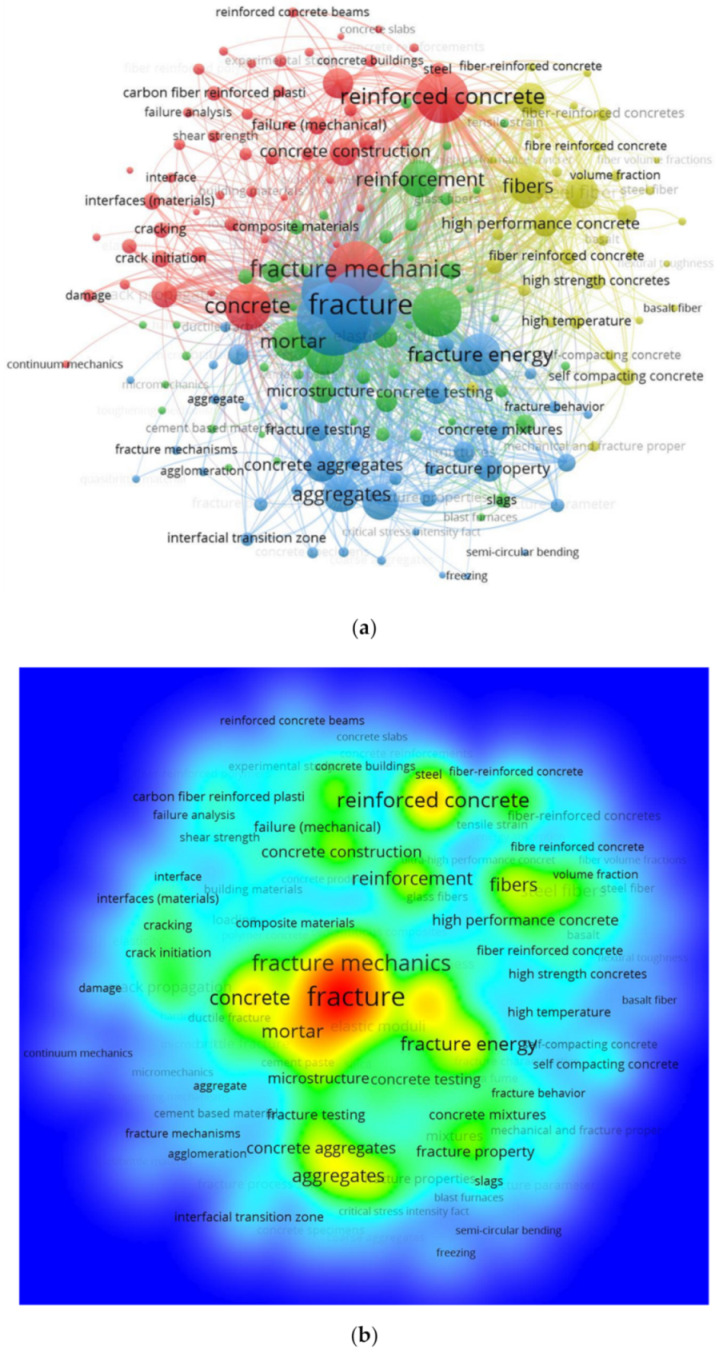
Network based on all keywords: (**a**) occurrence and (**b**) density.

**Figure 6:**
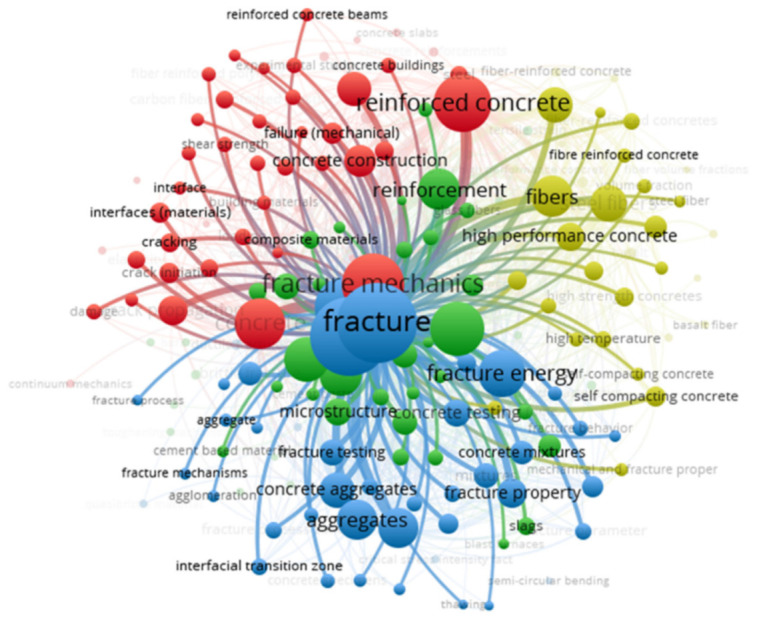
Fracture linkage with all factors.

Keywords comprise an important aspect of research that indicate and depict the fundamental area of a research domain [75]. The keywords used in this study that were found to have the most occurrences in the research articles are shown in Table 1. It was found that the top five most widely used keywords were concrete, reinforced concrete, fracture, fracture mechanics, and fracture energy. Figure 5 shows the keyword co-occurrence network, their visualization, connectivity to each other, and density corresponding to their link strength. The size of the keyword node represents the frequency of that particular keyword, while the keyword position represents its co-occurrence in publications. The visualization shows that fracture, fracture mechanics, and fracture energy have bigger nodes, thus demonstrating that these were found to be the most important keywords in the study of fracture properties. In the network, various keywords are identified by distinct colors that show keyword co-occurrence in various publications. Different colors show the various clusters of keywords, and it can be seen in Figure 5a that four clusters were identified (blue, red, yellow, and green). Specifically, the keywords with the most frequent co-occurrence—such as fracture, fracture energy, aggregates, fracture property, fracture testing, concrete aggregates, and interfacial transition zone—are shown by blue nodes. It was concluded that all these keywords have been repeatedly used in the publications on the fracture properties of FRCBMs. In the density visualization (depicted in Figure 5b), the higher and lower density keywords are identified with distinct colors. The order of colors is red, yellow, green, and blue, where red shows the highest density and blue shows the lowest. This finding will be helpful for keyword selection by authors to easily retrieve the published data in a particular domain in the future. The link of fracture with all the other keywords is shown in Figure 6. It is clear from the network that the word “fracture” is strongly linked to keywords such as fibers, aggregates, interfacial transition zone (ITZ), microstructure, and cracking.

### 3.3. Co-Authorship Scientific Mapping

**Figure 7:**
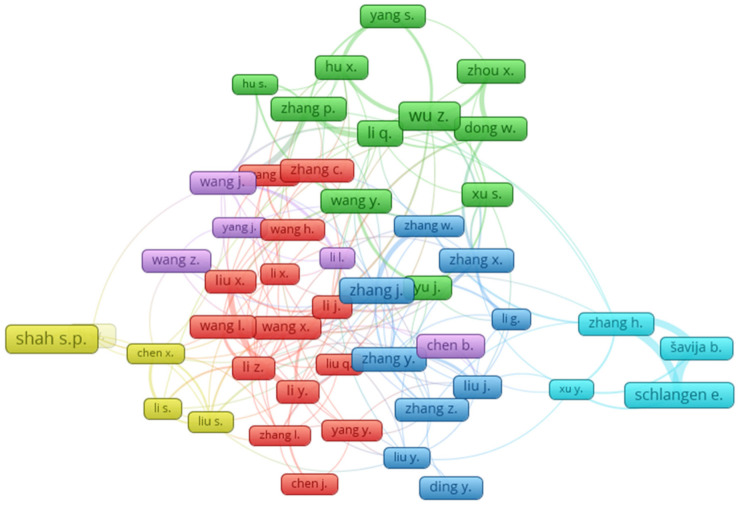
Researcher co-authorship (linkage based on citations).

The citation numbers of a researcher represent the level of influence of a researcher on a specific field [76]. Table 2 was generated to show the top 20 authors with the highest numbers of citations in the field of the fracture properties of FRCBMs, as retrieved from the Scopus database. Table 2 also indicates the highest number of documents published by an author in the subject domain, while the average citation number calculated by dividing the number of citations by the number of documents of each author. The maximum number of published documents was of Zhang J. (34 documents), while the highest number of citations was of Wu Z. (1398 citations). Quantitatively measuring an individual researcher’s efficiency would be hard. However, by comparing all the factors individually or with their synergy, the author’s ranking was possible. For example, as per the total citations, the top three ranked authors were found to be Wu Z. with 1398 citations, Shah S.P. with 1378 citations, and Elices M. with 1251 citations. Conversely, by comparing the average citations, the author’s rankings were found to be Shah S.P with 125 citations, Elices M. with 125 citations, and Planas J. with 107 citations. Furthermore, by comparing the number of documents, the ranking of authors were found to be Zhang J. with 34 documents, Li Q. with 28 documents, and Wu Z. with 26 documents. The most prominent authors’ linkage based on citations in the current study field is given in Figure 7. It is interesting to observe the linkage among the authors to contribute to the field of the fracture properties of FRCBMs.

### 3.4. Bibliographic Coupling Network Analysis

**Figure 8:**
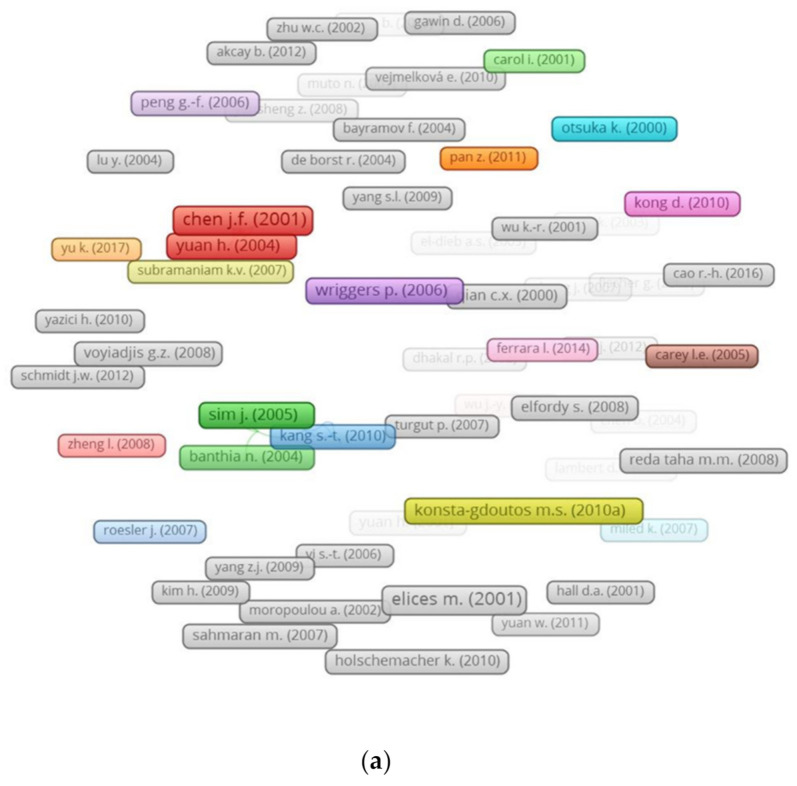

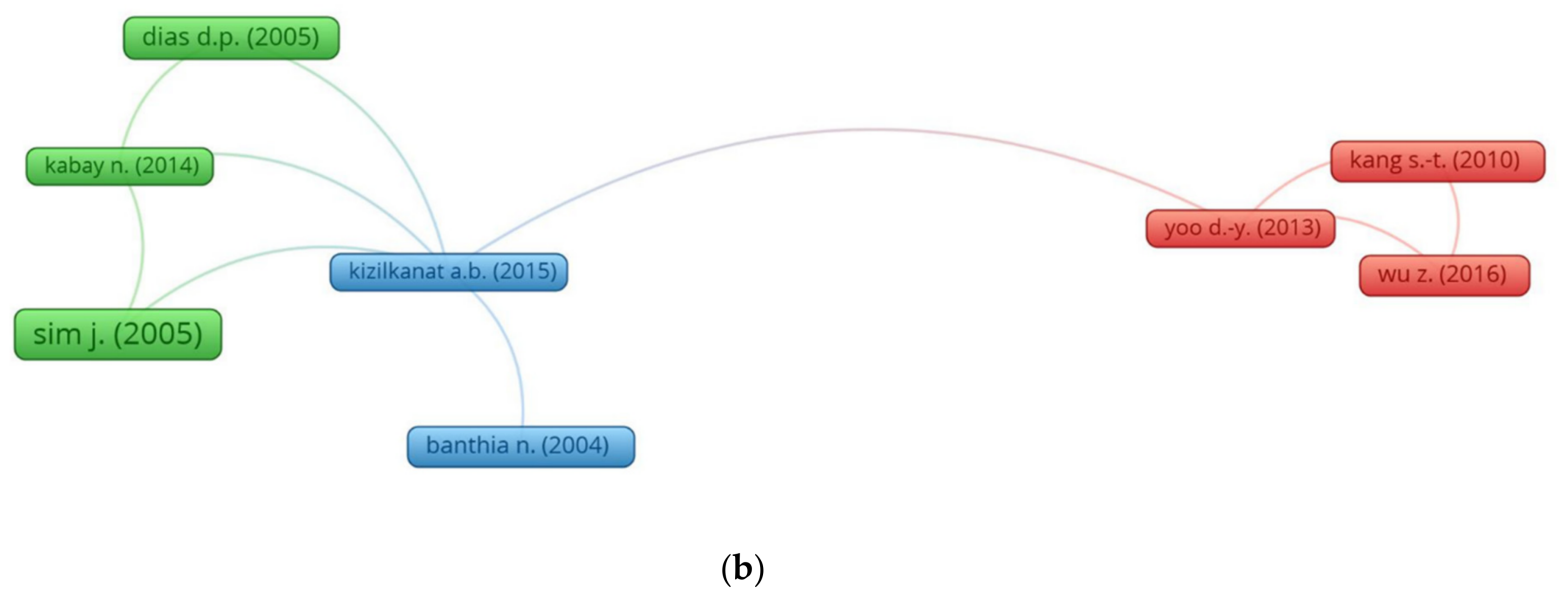
Authors linkage based on citations: (**a**) citations above 100 per article and (**b**) top connected articles.

The number of citations of a research article indicates the article’s impact on a specific research field. Articles with more citations could be considered milestones in the research field. Table 3 shows the most cited articles, their authors, and their publication year. Chen J.F. [77] was found to have the highest number citations, 851, on their article titled “Anchorage Strength Models for FRP and Steel Plates Bonded to Concrete.” However, Konsta-Gdoutos M.S. [10,78] was found to have two articles with citation numbers of 521 and 373, respectively, while Wu Z. [79,80,81] was found to have three articles with citation numbers of 266, 172, and 119, respectively. Figure 8 depicts a visualization of the authors with the most article citations, with a minimum of 100 citations, and the top connected articles in the present study field. The network revealed that most of the articles were not connected to each other based on citations. Only eight articles were found to have linkage with each other, as shown in Figure 8b, out of which only two articles by Yuan H. [82] and Kizilkanat A.B. [83] had the highest linkage number of five for each article.

### 3.5. Countries Active in Research of CBM Fracture Properties

**Figure 9:**
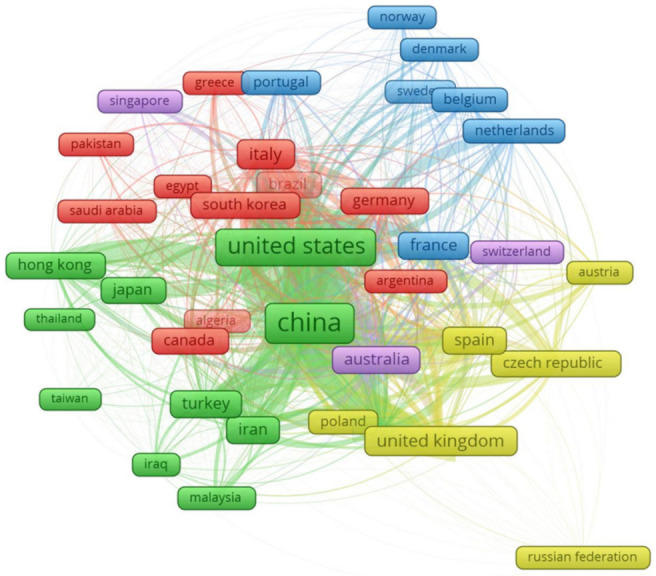
Country mapping.

The top 20 countries contributing to the research of CBM fracture properties are listed in Table 4. It was observed that the highest impact was of the United States, with 12,696 citations for 417 documents. China, United Kingdom, and Italy were found to have citation numbers of 12,487, 4002, and 3640, respectively, making them the most influential countries on the fracture properties of FRCBMs. The impact of a country in the growth of the current study domain is indicated by the number of documents, citations, and total link strength. The total link strength indicates the impact of documents from a country on other countries involved in these studies. China, United States, and Australia were the top three countries based on total link strength. The visualization network and density of countries based on citations are shown in Figure 9. The box size indicates the amount of contribution to the subject study area by the country. The graphical representation of contributing countries could help future scholars in developing research cooperation, creating joint venture studies, and exchanging innovative technologies and ideas.

## 4. Fibers

Fibers have been used in cement-based materials in the form of nanoparticles, whiskers, filaments, and threads to enhance the materials’ mechanical properties. For the selection of a fiber as reinforcement in a CBM, the main requirements to be considered are (i) material property compatibility with the applications, (ii) optimum aspect ratio to enhance post cracking behavior, and (iii) enough matrix–fiber interactions to transfer stresses. It is essential to illustrate the fiber material and geometric properties used in CBMs prior to discuss fibers and their combined action with CBMs.

### 4.1. Fiber Types

Compared to binders, fibers’ material properties are mostly more influential in modifying the properties of FRCBMs. For example, polypropylene (PP) fibers show weak binder–fiber contacts and decrease their composites’ performance, regardless of the binder type [96,97,98,99,100,101]. In this study, to report the essentials of material properties, the fibers that are the most used in CBMs were classified into five major groups explained in the following sub-sections. Table 5 summarizes the physical and mechanical properties of fibers.

#### 4.1.1. Steel Fibers

**Figure 10:**
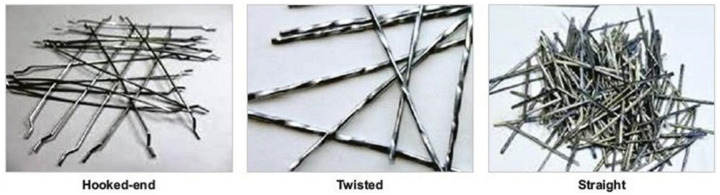
Steel fibers used in cement-based materials (CBMs). Reprinted from [116].

Steel fibers have been frequently used in CBMs because of their good strength, flexibility, and availability. As specified by ASTM A820-16 [108], for specific purposes, there are five types of steel fibers: (1) smooth or deformed cut sheet, (2) melt-extracted, (3) mill cut, (4) modified cold-drawn wire, and (5) pieces of smooth or deformed cold-drawn wire. These fibers are small enough to be easily spread randomly in CBMs. Depending on the fabrication process and material type, the tensile strength and ultimate elongation of steel fibers can vary from 310 to 2850 MPa and from 0.5% to 3.5%, respectively [109,110,111]. The bonding strength between a binder and steel fiber is higher than for other fiber materials because of the fiber’s corrugated surface [112,113]. However, despite some practical benefits, corrosion is a big problem with steel fibers [114,115]. To control the corrosion of steel fibers, these fibers are frequently used in the form of stainless steel alloys (ferritic, austenitic, duplex, martensitic, and precipitation hardening steels) and sacrificed coating composites (e.g., a zinc/copper coating) [112,113]. Based on longitudinal geometry, various types of steel fibers (i.e., hooked end, twisted, and straight) used in CBMs are shown in Figure 10.

#### 4.1.2. Polymeric Fibers

Polymers are basically made of long and repeating chains of molecules that are strongly bonded through intermolecular interactions [117]. Polymers have different properties because of differences in their intermolecular interaction. Polymers are classified on the basis of chain order as crystalline, semi-crystalline, or amorphous polymers [106,118]. Polymers with higher crystallinity values present higher mechanical properties, surface roughness, environmental stability, and rigidity properties. Moreover, depending on the material source and production process, polymeric fibers can be classified as synthetic or natural.

##### Synthetic Polymer Fibers

**Figure 11:**
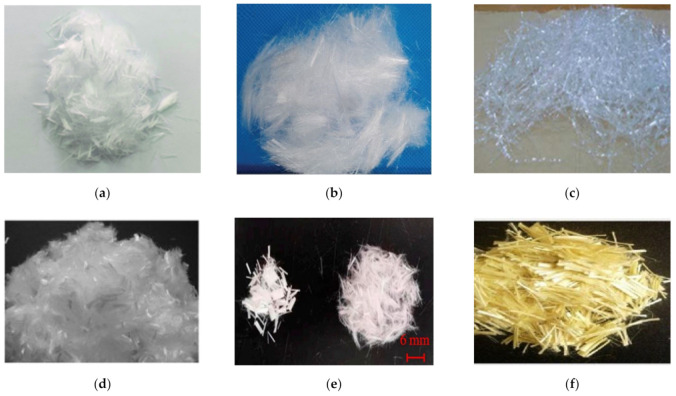
Synthetic polymer fibers used in CBMs. (**a**) Polypropylene [135], (**b**) Polyvinyl alcohol [136], (**c**) Polyethylene terephthalate [137], (**d**) Nylon [138], (**e**) Polyester [139], (**f**) Aramid [140].

These fibers are widely manufactured from raw ingredients or through plastic waste recycling. In the construction industry, the use of recycled fibers is a remarkable solution for the worldwide reuse of extensively used plastics such as PP and polyethylene terephthalate (PET) [117]. Various types of synthetic polymer fibers used in CBMs are shown in Figure 11. The commonly used synthetic fibers in CBMs include PP, PET, polyvinyl alcohol (PVA), and polyethylene (PE). PP is available with various shapes, sizes, and properties [119]. The major benefits of a PP fiber include its easy dispersion, low cost, control of the plastic shrinkage of CBMs, and inert behavior at high pH values [120]. However, it has a low modulus of elasticity, weak interfacial bonding, and a low thermal resistance because of its inherent hydrophobic nature [121,122,123,124]. Lately, fibers produced from recycled PET bottles have received major attention for engineering applications. PET fibers exhibit similar properties to those of nylon and PP fibers, but the production of these fibers is more environmentally friendly and less costly [125]. A PVA fiber presents a greater modulus of elasticity (29–42 GPa) and tensile strength (0.8–2.5 GPa). Furthermore, the presence of hydroxyl groups in the molecular chains of PVA fibers provides a good bonding ability with CBMs [126,127]. However, PVA is expensive compared to other synthetic polymers [128]. Moreover, a PVA fiber has a low capacity for fiber rupture due to its lesser lateral resistance that leads to the reduction of the the tensile strain capacity of FRCBMs [126,129,130]. PE fibers have variable properties depending on their crystallinity value, polydispersity, and molecular mass [131]. A high-density PE fiber has a tensile strength and an elastic modulus of up to 3.5 and 110 GPa, respectively [132]. However, this fiber has aquaphobic behavior [133,134].

##### Natural Polymer Fibers

**Figure 12:**
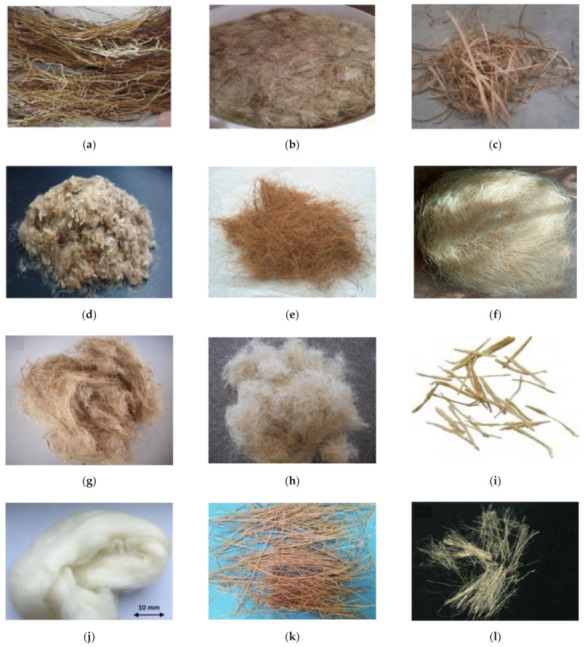
Natural polymer fibers used in CBMs. (**a**) Palm [144], (**b**) Hemp [145], (**c**) Banana [146], (**d**) Jute [147], (**e**) Coconut [148], (**f**) Abaca [149], (**g**) Kenaf [150], (**h**) Sisal [151], (**i**) Bagasse [152], (**j**) Wool [153], (**k**) Bamboo [154], (**l**) Flax fabric [155].

As an alternative to synthetic fibers, natural fibers like bagasse, hemp, jute, coconut, bamboo, wool, coir, banana, hemp, palm, and sisal can be used in CBMs, as shown in Figure 12. Natural fibers are preferred because these fibers are energy-efficient and made from environmentally friendly materials [141]. Additionally, natural fibers have further advantages like a wide availability, low cost, low thermal conductivity, reduced density, and improved mechanical properties. However, there are several disadvantages of natural fibers, including (i) poor interactions with matrixes, (ii) inconsistency in material properties, (iii) durability issues, and (iv) reductions in the workability of fresh mixes at higher fiber contents [141,142,143].

#### 4.1.3. Inorganic Fibers

**Figure 13:**
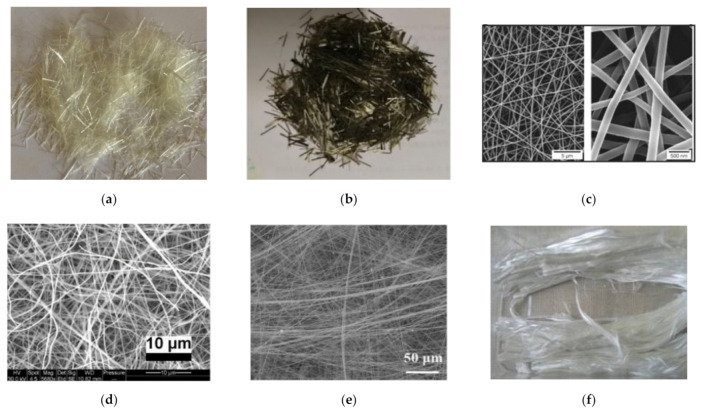
Inorganic fibers used in CBMs. (**a**) Glass [83], (**b**) Basalt [19], (**c**) Boron nitride [165], (**d**) Alumina [166], (**e**) Silicon carbide [167], (**f**) Asbestos [168].

The use of inorganic fibres such as asbestos can be seen in ancient times [156]. Inorganic fibers comprise silica and alumina mixtures, and their melting point is very high, thus allowing them to have wide applications at elevated temperatures, e.g., furnace linings. Additionally, these fibers exhibit good tensile strength, excellent insulation, good chemical stability, and low cost [102]. The most used inorganic fibers in CBMs are described below and shown in Figure 13.

##### Silica Fibers

These are metal oxide fibers having a higher value of SiO_2_ than other inorganic fibers. These fibers are further classified commercially as structural glass (S-glass), alkali-resistant glass (AR-glass), chemical glass (C-glass), and electrical glass (E-glass). Due to the high alkaline environments of CBMs, various glass fibers are susceptible to decay [157]. To survive the alkaline environments of CBMs, AR-glass has been produced and used [107,122].

##### Aluminosilicate and Alumina Fibers

These are also metal oxide fibers comprising 45–60% Al_2_O_3_ and silicates. These fibers are manufactured by the spun or blown methods of melted kaolin or associated precursors/clays, which include Al_2_O_3_ and SiO_2_. The mechanical properties of these fibers depend on the aluminate to silicate ratio. With increasing alumina contents, their resistance to higher temperatures increases, but at higher silica contents, their tensile strength is higher and their elastic modulus decreases [102]. Additionally, the thermal shrinkage and tensile strength of these fibers can be affected by their amorphousness. For example, when the crystallinity of aluminosilicate fibers was increased from 50% to 100% at 1400 °C, their tensile strength and shrinkage were reduced from 1800 to 500 MPa and from 18% to 0%, respectively [158].

##### Basalt Fibers

Basalt fiber is a broadly used inorganic fiber in CBMs that originates from volcanic rocks melted at a high temperature of 1500–1700 °C [159]. Basalt fiber has the advantages of low cost, wide availability, durability, high strength, and high heat resistance. Additionally, it is exceptionally hard and has excellent resistance to abrasion [160]. In addition, it was found that after corrosion in alkaline conditions, these fibers exhibit significant resistance in acidic environments [161]. The temperature limit at which basalt fiber is applicable ranges from 200 to 800 °C. However, at higher temperatures, basalt fiber may experience structural changes [162].

##### Other Inorganic Fibers

There are numerous other inorganic fibers with better mechanical and thermal properties that are being used in CBMs for specific applications, including silicon nitrite, silicon carbide, zirconia, boron, boron nitride, boron carbide, and different whiskers. Reviewing these fibers is a worthy task, but it was beyond the current study scope. However, further details are available in the literature [102,163,164].

#### 4.1.4. Carbon Fibers

**Figure 14:**
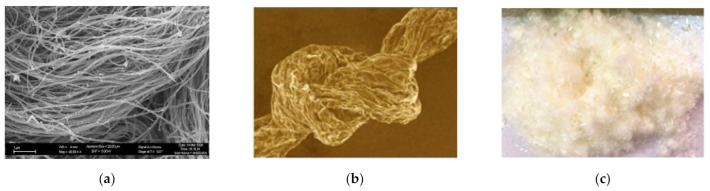
Carbon fibers used in CBMs. (**a**) Carbon nanotube [185], (**b**) Graphene [184], (**c**) Polyacrylonitrile [186].

Carbon fiber is comprised of carbon atoms connected together in the form of a long chain. These fibers have excellent tensile strength, light weight, high thermal and electrical conductivity, less thermal expansion, chemical stability, and thermal stability [169,170]. Additionally, these fibers are extremely elastic, and they are less affected by fatigue deformation during the loading–unloading cycle [171,172]. Carbon fibers are classified according to geometry into two main groups: carbon nanofibers and uniform length fibers. Additionally, according to tensile modulus, carbon fibers are classified into low modulus, standard modulus, intermediate modulus, high modulus, and ultra-high-modulus groups [173]. The various types of carbon fibers that are commonly used in CBMs are shown in Figure 14.

##### Polymeric Carbon Fibers

Polymeric carbon fibers are produced by the carbonization process and primarily originate from sources like petroleum pitch, rayon, and polyacrylonitrile (PAN) [105]. Amongst these, PAN fibers are mostly commercially used (about 90%) due to the stability in their tensile strength and production cost [174]. Their tensile strength and tensile modulus range from 2.5 to 7.0 GPa and from 250 to 400 GPa, respectively [105]. Compared to PAN-based fibers, pitch fiber, which is an oil refinery residue, has a reduced tensile strength (1.5–3.5 GPa) and a greater tensile modulus (up to 900 GPa) [175]. Rayon-based fibers have a low tensile modulus ranging from 35 to 60 GPa, which makes them less favorable and mostly used in low thermal conductivity applications [176]. These fibers can also be classified based on their tow size. For example, less than 24,000 tows are termed regular tows, whereas greater than 50,000 tows are called large tows [173].

##### Carbon Nanofibers

Carbon nanofibers are produced in the whisker form with a diameter of about 0.5–1.5 μm or even less. Carbon nanotubes (CNTs) are commonly used nanofibers [177]. These are manufactured in two forms, i.e., single-wall carbon nanotubes (SWCNTs) and multiwall carbon nanotubes (MWCNTs). Their tensile strength and young’s modulus range from 11 to 63 and from 1000 to 1800 GPa, respectively [178,179]. SWCNTs are more flexible than MWCNTs, but their tensile strength is lower than that of MWCNTs [178]. Graphene has an extremely high aspect ratio, due to which it is used as reinforcement in CBMs, even though it is not a fiber [180,181,182,183,184]. Thus far, various graphene types, like graphene oxide (GO) and reduced graphene oxide, have been effectively manufactured. Amongst these, GO is highly oxidized in nature and exhibits higher flexibility and performance than reduced graphene oxide [106].

### 4.2. Fiber Geometry

The length, cross-section, fiber surface area, and fiber cross-sectional area along the fiber length are the most important fiber geometrical parameters that need to be considered in fiber efficiency evaluation.

#### 4.2.1. Size of Reinforcement

**Figure 15:**
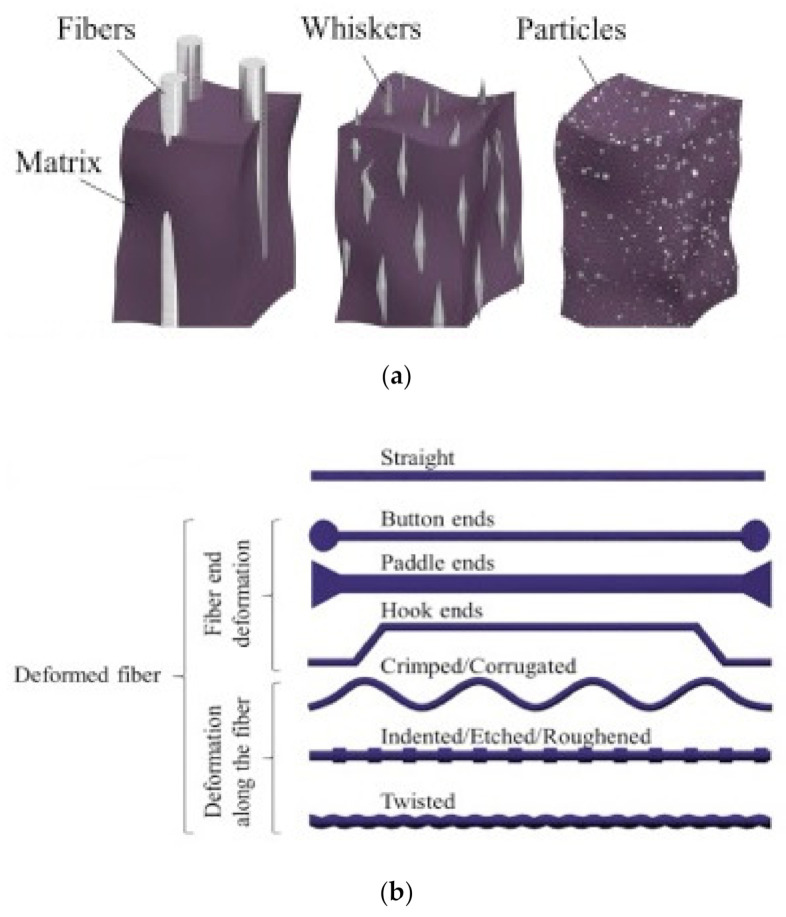
Characteristics of (**a**) types of reinforcements based on particle size and (**b**) fiber longitudinal geometry. Reprinted with permission from [193]. Copyright 2021 Elsevier.

Reinforcements can be of three types: fibers, particles, and whiskers, as shown in Figure 15a. When the fiber diameter increases, its mechanical strength and modulus decrease [102,187]. This can be clearly noticed in alumina fiber, inorganic materials [187], polycaprolactone [188], glass fiber [189], PVA fibers [126,190], drawn wires, and inorganic whiskers [102]. This may be due to the fact that the possibility for defects and imperfections are higher in large-diameter fibers than that for small diameter fibers or single-crystal whiskers [102,163].

#### 4.2.2. Cross Section and Longitudinal Geometry

Very few fibers are individually manufactured in geometric forms. To improve the fiber–binder bonding, it is desirable to pre-deform the fibers during the production process [191,192]. The ends of fibers can be deformed into shapes such as hooks, buttons, and paddles, and the longitudinal section can be deformed by twisting or crimping fibers, as shown in Figure 15b. Additionally, fiber cross-sections vary in a wide range of shapes such as rounded, prismatic, and polygonal with corrugated and smooth surfaces, as well as uneven cross-sections, variable lengthwise cross-sections, multifilament networks, and monofilament networks.

#### 4.2.3. Equivalent Diameter

This is the measure of the diameter of non-circular fibers. An equivalent diameter shows the average cross-sectional area of the real fiber.

#### 4.2.4. Efficient Diameter

The efficient diameter is the smaller diameter of a fiber along its length, which indicates the best load resisting capability of that fiber.

#### 4.2.5. Fiber Aspect Ratio

The fiber aspect ratio is the ratio of fiber length to the equivalent diameter of the same fiber. This represents fiber slenderness. For short FRCBMs, this value ranges from 40 to 2000, but it is mostly below 300.

#### 4.2.6. Fiber Count and Specific Surface

Cracks develop across fibers due to the consumption of energy, which is based on the fiber quantity/count confronted and the surface area. For example, the pull-out property depends on the cross-sectional area of the fibers inside the crack plane, while debonding depends on the surface area of the affected fibers.

## 5. Factors Affecting Fracture Properties of FRCBMs

FRCBMs usually comprise two phases, i.e., matrix phase and dispersion phase, and both phases affect their mechanical properties. The fracture properties of FRCBMs depend on the fiber properties (e.g., type, content, shape, and size) and cement matrix properties (e.g., water–cement ratio, aggregate size, and aggregate type).

### 5.1. Influence of Fiber

The most significant factor considered for the changing the failure mode of CBMs is the addition of fibers. As previously mentioned in Section 1, fiber addition to CBMs as reinforcement can improve the various properties of a cement matrix. Table 6 summarizes the various fiber types used to study the fracture properties of FRCBMs.

#### 5.1.1. Fiber Type

The fracture process usually starts at the micro level because of the multi-scale behavior of CBMs [11]. The stress concentration near micro-cracks is the main cause of fracture failure. MWCNTs can remarkably enhance the fracture properties of CBMs. MWCNTs have the exceptional capability to provide toughness because these fibers exhibit exceptional mechanical and chemical properties and a high aspect ratio [9]. Furthermore, the modification of MWCNTs can further improve mechanical properties. For example, cement mortar reinforced with MWCNTs was found to enhance fracture toughness by 86% compared to plain mortar. After the modification of MWCNTs, the result for fracture toughness was 125% higher than that of plain mortar. This may have been due to the excellent interfacial bonding of MWCNTs with the cement matrix [206]. A calcium carbonate whisker (CW) is used in cement paste as reinforcement and has been shown to improve the energy absorption and crack resistance ability of composites. Well-dispersed CWs in a matrix could efficiently delay micro-crack development and propagation, as well as improve mechanical properties [207]. Additionally, GO addition in concrete can improve fracture toughness because GO is highly reactive and hydrophilic, and it might induce active group hydration in cement [195]. Fibers (such as MWCNTs, CW, and GO) show valuable microscopic crack bridging, interfacial improvement, and filler action in matrixes. GO fibers and MWCNTs exhibit other nucleation effects because these micro-fibers have the ability to facilitate the hydration of cement [11,194,195]. The multi-scale fracture and mechanical characteristics of CBMs can be improved by uniformly distributed MWCNTs and carbon nanofibers (CNFs) [10,78]. However, it is hard to disperse these fibers uniformly in a matrix due to Van der Waals forces. Methods for the uniform dispersion of micro-fibers in matrixes include the ultra-sonication and chemical treatment of these fibers. A study on the comparison of fracture properties of cement paste was carried out by Hu et al. [208] using dispersed treated and undispersed MWCNTs. It was reported that dispersed treated MWCNTs led to a remarkable improvement in fracture properties. Liu et al. [194] subjected MWCNTs to ultrasonic treatment, and the results revealed a significant increase in fracture properties of cement paste. Furthermore, Luo et al. [195] used a high-range water reducer based on a polycarboxylate polymer as a dispersant for GO.

The most common and extensively used fiber in research, as well as engineering applications, to improve the performance of CBMs is steel fiber. Steel fibers’ mechanical properties have a substantial influence on the fracture behavior of FRCBMs. One of the most important factors is the tensile strength of steel fiber, which has a direct effect on fracture properties and should be considered in steel fiber-reinforced concrete (SFRC) design. For instance, when higher tensile strength steel fibers were used in CBMs, keeping the water–cement ratio (w/c) constant, the fracture properties were found to be further improved [24]. Additionally, the fracture properties of FRCBMs depend on the type of steel fiber (such as short, long, straight, and hooked end). Short and straight steel fibers are not as efficient as hooked end fibers in improving the fracture properties [209]. One factor that plays a vital role in the structural behavior of CBMs is the development and progress of the fracture process zone. The inclusion of steel fibers in cement mortar can control the development of the fracture process zone, resist crack creation, and ultimately decrease structural member sizes [210].

A chemically aggressive environment may affect the durability of composite-reinforced with steel fibers. Additionally, the problems of electric and magnetic fields may have adverse effects [8]. Consequently, as alternative reinforcing materials, synthetic fibers have wide applications due to several advantages over steel fibers, including low cost, chemical stability, and minute electromagnetic interference [8,203,211,212,213]. The most commonly used synthetic fibers are PP, PVA, and polyolefin fibers. When polyolefin fibers were added to self-compacting concrete (SCC), the composite was found to achieve similar fracture properties to those of steel fibers, while the fiber content was lesser in terms of weight than that of steel fibers [214]. The SCC reinforced with polyolefin fibers had higher fracture properties and better ductile behavior [213,214]. PP fibers, with a lower modulus, are more broadly used than polyolefin fibers for reducing shrinkage in concrete and preventing explosive spalling [203,213].

In addition to synthetic and steel fibers, various inorganic mineral fibers like glass, carbon, basalt, and brucite fibers are used as reinforcements in CBMs. Basalt and glass fibers have comparable mechanical properties, but basalt fiber has the benefits of higher strength, higher elastic modulus, lower cost, longer term durability, better chemical stability, and better thermal stability than glass fiber [215,216]. Arslan [205] reported that the incorporation of glass and basalt fibers into concrete led to more enhanced fracture properties than PC. Kizilkanat et al. [83] conducted a comparison on the addition of glass and basalt fibers in concrete and found that compared to glass fiber, basalt fiber is more able to enhance the fracture parameters of CBMs. The addition of carbon fibers in high-strength concrete (HSC) was found to significantly improve the concrete’s fracture properties and load-bearing capacity [217].

Presently, the previously mentioned fibers (synthetic and inorganic) are used in CBMs as conventional reinforcement materials, but a high amount of energy is consumed in their manufacturing processes and has become a source of heavy pollution. Natural fibers are used as alternatives because these are produced in a more ecological mode and can be reused/recycled. Hence, the addition of natural fibers as reinforcement in CBMs has progressively gotten more attention in civil engineering applications [218]. Natural fibers have a poor resistance towards alkali attacks, which is the main disadvantage of these fibers. However, several techniques (like the delignification process) are available and can be used to ensure the stability of these fibers in an alkaline environment. The delignification process has been conducted for banana, eucalyptus, and sisal fibers for reducing the lignin content of these fibers and to enhance ductility. Merta et al. [218] improved hemp fiber durability against alkali attacks by using linseed oil with a catalyst as a protective material to provide water resistance to the fiber. The type of used natural fiber greatly affects the mechanical properties of FRCBMs. Furthermore, several agricultural wastes like sugarcane bagasse, banana, and coconut fibers have been used in CBMs to improve their fracture properties [219]. In conclusion, several types of fibers have been used in civil engineering applications, e.g., steel fibers for pavements, non-structural members, and tunnel lining [220]; basalt fibers as reinforcement in roads and stucco nets [216]; and PVA fibers for engineered CBMs [39].

The aim of fiber hybridization is to attain the best result of each contributing fiber at various levels in cement paste, mortar, and concrete [220,221,222]. There are various methods of fiber hybridization. One of them, as reported by Banthia [223], is the combination of distinct sizes/lengths of fibers. Hybridization with different sizes/lengths of fibers performs its role as reinforcement at different scales. Uniformly distributed short fibers in a matrix control the formation and expansion of micro-cracks, though long fibers resist macro-cracks via the bridging effect [5,12,20,24]. Rasheed et al. [224] used mixed micro and macro synthetic fibers in cellular lightweight concrete to improve fracture properties. It was found that the fracture load was increased by 34% with the addition of 0.02% micro and 0.4% macro-fibers. It was reported by Long et al. [209] that the hybridization of steel fibers enhanced fracture energy by up to 37.5 times and fracture toughness by 29% compared to PC. Additionally, micro-fibers can decrease porosity and increase toughness in a matrix.

**Figure 16:**
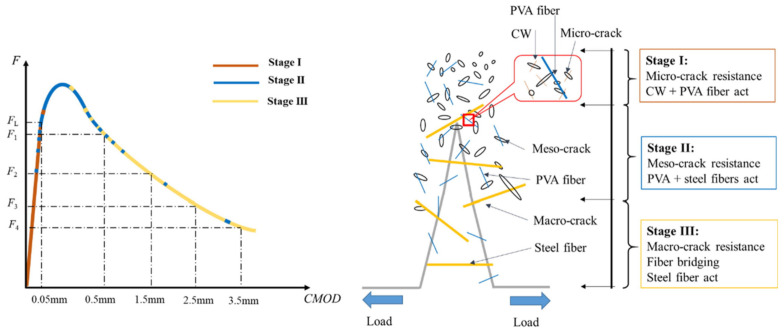
Influence of hybrid fibers during crack propagation [225]. CW: carbonate whisker; PVA: polyvinyl alcohol; CMOD: crack mouth opening displacement.

Another method of fiber hybridization is the combination of fibers with different moduli. The combination of steel and PP fibers could enhance the fracture parameters of CBMs due to the synergetic effect among both fibers during fracture [223]. Related studies have also stated a synergy on fracture toughness at small deflections with the hybridization of large steel fibers (hooked end) and PP fibers. The synergy effect reduces with increasing deflection [220]. A PP and basalt fiber combination in HPC was found to control crack width and improve fracture properties [28]. In another study by Cao et al. [33], CW–PVA–steel hybrid fibers resisted cracks at several phases throughout crack growth, as shown in Figure 16. At stage I, resistance to the micro-cracks was provided by the combined action of the CW and PVA fibers. At stage II, the PVA and steel fibers restricted the meso-crack propagation and played a role in bridging the extended cracks after the peak. In stage III, steel fibers were more effective in providing resistance to macro cracks after peak load due to their longer length. Thus, it was concluded that fiber type is an important factor that can affect the fracture parameters of cementitious composites, and various types of fiber are effective at various levels due to their scales and sizes.

#### 5.1.2. Fiber Content

The key function of fiber addition is to resist the development of micro-cracks and to improve the mechanical properties of CBMs. Increasing fiber content within a suitable range can improve fracture properties due to the fact that more fibers will be available to resist cracks. Hu et al. [208] reported an increase of 6.3% in fracture toughness and an increase of 21.5% in fracture energy when cement paste was reinforced with 0.05% MWCNTs. When the MWCNT content increased to 0.1%, the fracture toughness and fracture energy further increased by 11.4% and 26.2%, respectively. Meanwhile further increments in fiber content caused the reduction of fracture properties due to the non-uniform mixing and agglomeration of fibers in the matrix. The research results of Gdoutos et al. [9] revealed that at a low content (i.e., 0.1%) of MWCNTs, fracture properties were significantly improved, while at a higher content (i.e., 0.2%), the same parameters showed slightly decreased values. This may have been due to the improper mixing and agglomeration of MWCNTs in the matrix, which produced a local stress concentration zone and ultimately reduced the fracture properties of the FRCBM.

Similarly, Kazemi et al. [34] reported that due to an increase in fiber content, the fracture properties increased and resulted in a more ductile performance of SFRC. The increase in fracture properties with increasing fiber contents has been reported by many other researchers [24,34,196,197,201]. Similar to the steel fibers, the addition of various other types of fibers (e.g., PP fiber [7,226], glass, and basalt fiber [83]) had also been found to result in the enhancement of the fracture properties of CBMs due to the increase in fiber content. However, there is a critical value for the fiber content of all fiber types, beyond which there will be a reduction in fracture properties of FRCBMs because the excess fibers are difficult to uniformly distribute in a matrix, which results in the agglomeration or balling of fibers. Cao et al. [226] reported that PP fiber content of 3.2% in concrete resulted in a lower cracking toughness than with 1.6% PP fiber content. It was also reported by Arslan [205] that fracture energy slightly decreased after the glass fiber content exceeded 1 kg/m^3^ and the basalt fiber content exceeded 2 kg/m^3^. Hence, it was revealed that fiber content is an important parameter that affects the properties of CBMs, and the optimum contents of fibers are beneficial for enhanced fracture parameters. Beyond the optimum content, fracture properties can be decreased due to an excess amount of fiber that results in a non-uniform and heterogeneous mix.

### 5.2. Influence of Cement Matrix

Concrete is a heterogeneous material that has three primary phases: the cementitious matrix, aggregates, and the interfacial transition zone. The complicated nature of concrete is based on the behavior of these three phases. In other words, knowledge of the cementitious matrix, aggregates, and ITZ is critical to understand the interrelationships between concrete phases, structure, and properties. The ITZ mostly depends upon the water to cementitious (w/c) ratio of concrete [227]. Failure happens and spreads through the ITZs of dissimilar material-matrixes and aggregates [228]. Researchers have also discovered that the type of aggregate used in concrete has an impact on its properties [229,230].

#### 5.2.1. Influence of Water–Cement Ratio

The addition of fibers to a CBM produces more interfaces in the matrix compared to PC. The w/c is a major factor that affects the fracture behavior of an FRCBM because it can change the ITZ and pore structure of the composite. The defects in a matrix can be reduced at a lower w/c, which can further improve the fiber–matrix ITZ. Mostly, fracture properties improve with lower w/c values [24,33,231,232], as well as tensile strength of fiber [197,231]. Usually, the energy consumption of FRCBMs mostly occurs through fiber fracture and fiber pull-out from the matrix, depending on the strength of the binder. When a high strength binder is used, fiber fracture will occur; when using a low-strength binder, fiber pull-out will occur. However, the materials are not completely utilized to increase the energy consumption of FRCBMs in both cases. Thus, to achieve maximum energy consumption, the fiber–matrix ITZ must be improved so that fiber pull-out and fiber fracture occur at the same time.

#### 5.2.2. Influence of Aggregate

##### Aggregate Maximum Particle Size

An essential ingredient of concrete is aggregate, which is a filler and skeleton of the matrix. The fracture parameters of FRCBMs can be affected by the maximum aggregate size (d_m_) to some extent because within the matrix, cracks will break or bypass the aggregate [196,197,232]. Fracture properties improve more with a smaller d_m_ than with a larger d_m_, though the consumption of energy of broken aggregate or bypass distance of crack is higher at a large d_m_. The effect of a larger d_m_ and steel fiber together was shown to result in an better fracture toughness than that of a material with a smaller d_m_ and steel fiber. After increasing the d_m_ in SFRC, the fracture toughness was found to be improved [232]. Additionally, due to the increase in d_m_, the fracture energy of SFRC was found to be improved. However, the effect of a large d_m_ on fiber dispersion in a matrix must be considered. Generally, it is obvious that the fracture performance of an FRCBM mostly depends on fibers in the matrix instead of aggregate. Due to the availability of a large d_m_, the available space for fiber uniform dispersion becomes smaller in a matrix, which may lead to decreased FRCBM fracture parameters. In addition to crack resistance, an optimum d_m_ also enhances the distribution and orientation of fibers in a matrix and ultimately enhances the ductility and energy absorption capability of an FRCBM [196]. Ghasemi et al. [196] studied the combined effect of the steel fiber content and d_m_ on fracture energy of FRCBMs. It was found that increasing the d_m_ up to 12.5 mm enhanced the fracture energy absorption of the matrix, while a further increase in the d_m_ resulted in reduced energy. Therefore, the use of an optimized maximum aggregate size can result in better fracture properties.

##### Aggregate Type

Recently, in the analysis of the fracture performance of FRCBMs, various new types of aggregates like lightweight aggregates [233], recycled aggregates [202], and rubber particle [201,234] have appeared in addition to conventional aggregates (pebble, rock, etc.). The various aggregate types may have different chemical compositions, strengths, and elastic moduli, which may affect matrix properties. Hence, aggregate type has a great influence on FRCBM performance. Lightweight aggregate concrete has been widely used in structural applications due to its light deadweight. Guneyisi et al. [233] reported that lightweight aggregate steel fiber-reinforced concrete showed better fracture properties at 45% lightweight aggregate compared to that at 60% due to the fact that the strength of lightweight aggregate was less than the cement matrix. Currently, for green construction, materials are being recycled from dismantled buildings for utilization in concrete. Similarly, industrial and agricultural solid waste are also recycled and reused [201,202,234]. Recycled rubber aggregate can increase concrete’s energy absorption capacity. Noaman et al. [234] replaced natural sand with crumb rubber aggregate in FRCBMs. It was reported that crumb rubber aggregate with steel fibers enhanced the fracture energy from 190% to 246%, when rubber content increased from 5% to 20%, respectively. However, an increase of crumb rubber aggregate content in FRCBMs was found to result in a decreased fracture toughness because the compressive strength was reduced with the addition of the crumb rubber. Furthermore, Xie et al. [201] and Guo et al. [202] examined the effect of the addition of recycled or crumb rubber aggregate on the fracture properties of FRCBMs, and it was found that various types and sizes of aggregate significantly improved the fracture characteristics of the FRCBMs.

## 6. Analytical Models on Fracture Properties

In addition to the experimental work, researchers have presented analytical/constitutive models to predict the properties of FRCBMs [33,53,235,236]. Cao et al. [33] performed experimental work and regression analysis to develop analytical models to predict the fracture parameters of hybrid fiber-reinforced CBMs and made a comparison. As the characteristic parameters of hybrid fibers, the comprehensive reinforcing index (RI_v_) was introduced. The calculation of FRCBM fracture parameters was proposed by empirical formulas while considering both fiber (RI_v_) and matrix (w/c) variables. The established models for calculating fracture parameters are presented by the equations below:

(a) Initial fracture toughness ($K_{IC}^{ini}$) model

$$K_{IC}^{ini}\;=\;-27.7{\left(RI_{v}\right)}^{2}+40.2{\left(RI_{v}\right)}^{2}\left(w/c\right)-15.7\left(RI_{v}\right)\left(w/c\right)+10.9\left(RI_{v}\right)-2.7\left(w/c\right)+1$$

(b) Unstable fracture toughness ($K_{IC}^{un}$) model

$$K_{IC}^{un}=-361{\left(RI_{v}\right)}^{2}+416{\left(RI_{v}\right)}^{2}\left(w/c\right)-177\left(RI_{v}\right)\left(w/c\right)+153.7\left(RI_{v}\right)-1.4\left(w/c\right)-0.9$$

(c) Fracture energy ($G_{F}$) model

$$G_{F}=-297288.2{\left(RI_{v}\right)}^{2}+617011.4{\left(RI_{v}\right)}^{2}(w/c)-298328.1\left(RI_{v}\right)(w/c)+143740.3\left(RI_{v}\right)+2248.1(w/c)-1600.8$$

(d) Length of fracture process zone ($\Delta a_{c}$) model

$$\Delta a_{c}=-235.8{\left(RI_{v}\right)}^{2}-253.3{\left(RI_{v}\right)}^{2}(w/c)+109.2\left(RI_{v}\right)(w/c)+101.7\left(RI_{v}\right)+2.2(w/c)+1.1$$

**Figure 17:**
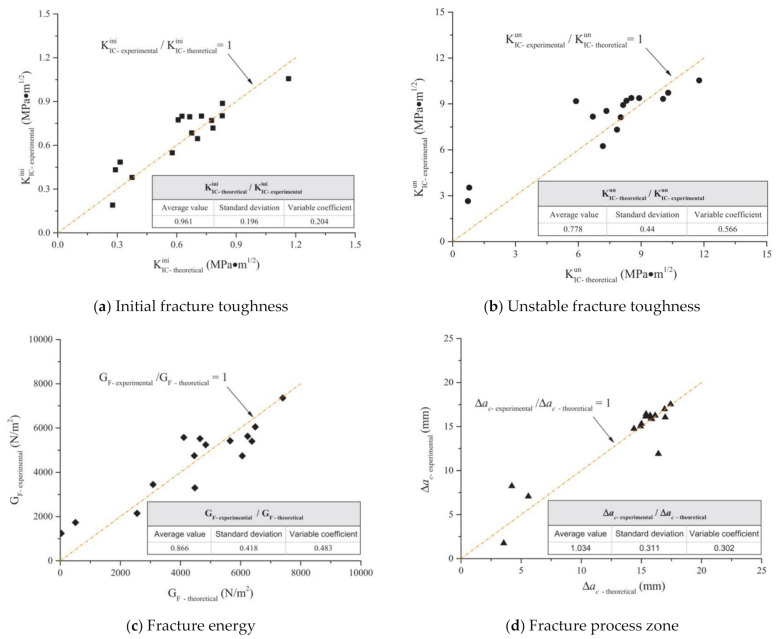
Relationship between theoretically and experimentally obtained fracture parameters. Reprinted with permission from [33]. Copyright 2021 Elsevier.

The relationship between theoretical fracture parameters calculated using the above equations and experimental results is shown in Figure 17. The theoretical and experimental findings were found to be in good agreement.

## 7. Discussions on Fracture Mechanism

**Figure 18:**
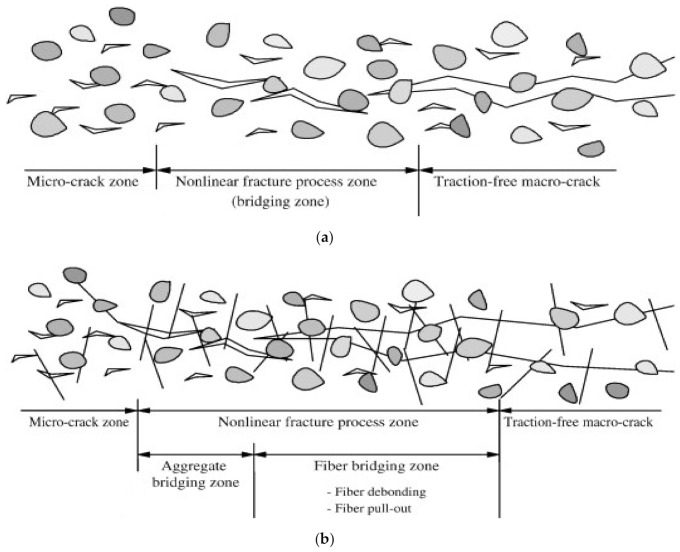
Mechanism of crack resistance: (**a**) plain concrete (PC) and (**b**) FRCBMs. Reprinted with permission from [237]. Copyright 2021 Elsevier.

The crack-resisting mechanism of FRCBMs for fracture performance is not the same as that of PC because of the availability of randomly distributed fibers in the matrix, which has a great effect on fracture process zone and ductility. In PC, the occlusal effect of aggregates dominates fracture behavior, while in FRCBMs, both the aggregate and fiber bridging occlusal effects should be considered (Figure 18) [237].

**Figure 19:**
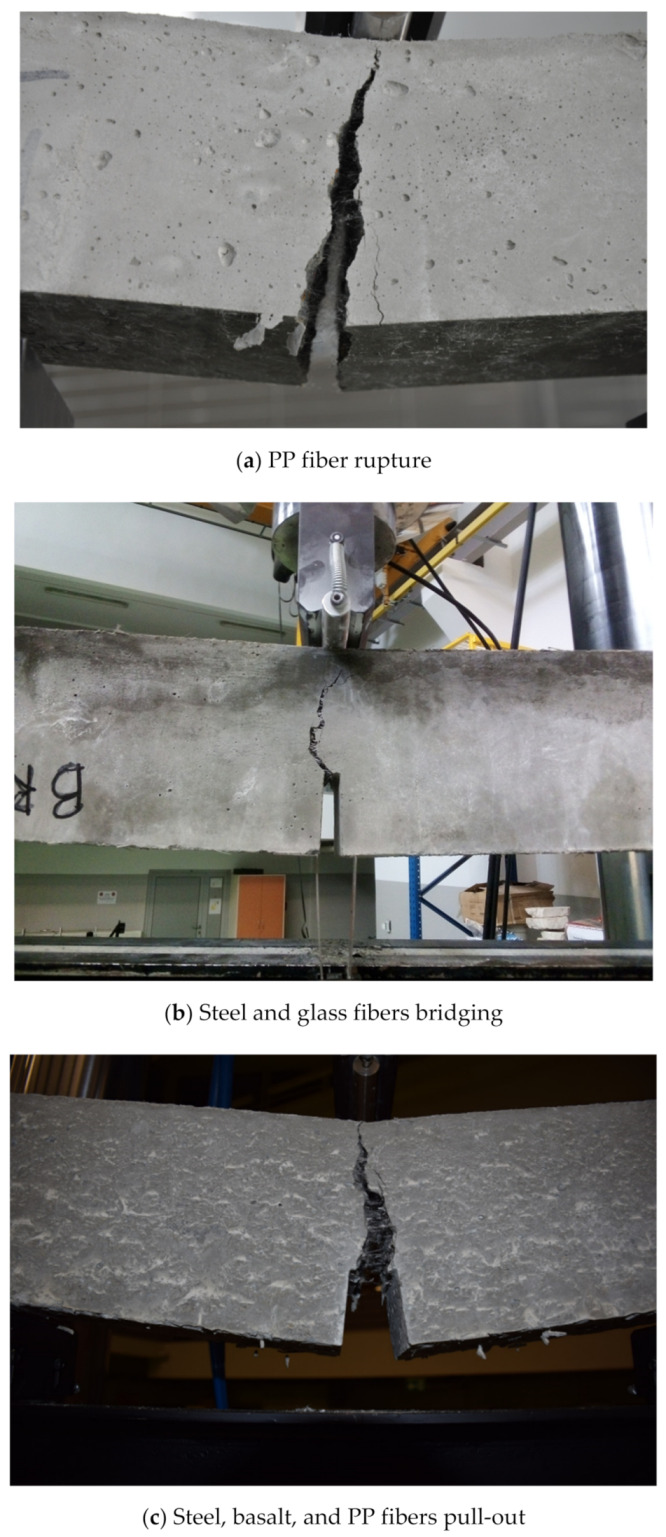
Crack-resisting mechanism of fibers during fracture from an ongoing research work of authors.

**Figure 20:**
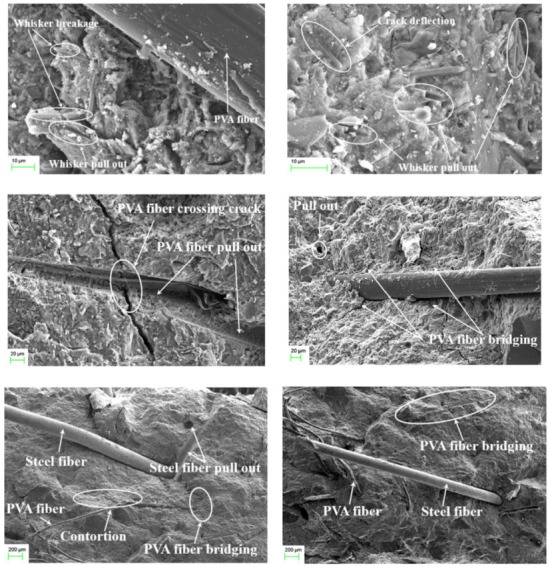
Fiber rupture, fiber bridging, and fiber pull-out of hybrid fibers in CBM matrixes obtained by SEM. Reprinted with permission from [33]. Copyright 2021 Elsevier.

The main crack-resisting mechanism of FRCBMs include fiber rupture, fiber bridging, and fiber pull-out (Figure 19). Fiber ruptures, fiber bridgings, and fiber pull-outs of various types of fiber are shown in Figure 20. Sahin and Koksal [231] reported that fiber rupture and fiber pull-out directly affect the fracture performance of FRCBMs, and crack bridging performance depends on fiber pull-out. Therefore, the important factor in enhancing ductility is better fiber–matrix bonding. Carpinteri et al. [7] also illustrated the importance of fibers in bridging cracks and resisting crack propagation due to the interfacial debonding of the fibers and the matrix. Furthermore, Cifuentes et al. [203] stated that the most important process to enhance the fracture energy in FRCBMs was fiber pull-out in the case of normal or low strength concrete, while fiber rupture or breaking was most important in HSC.

However, the crack-resisting mechanism of micro-fibers in CBMs to enhance fracture performance is not limited to fiber pull-out, fiber bridging, and fiber rupture/breaking. The addition of fibers can also enhance the ITZ and composite properties. Liu et al. [194] found that well-dispersed MWCNTs embedded in cement hydration products could have the ability to develop a strong bond and form a web-like dispersion for bridging pores/defects. An improvement in the mechanical properties of cement paste was found due to better stress transfer between MWCNTs and the cement matrix. Similar behavior and conclusions were also reported by Hu et al. [208].

Similarly, uniformly distributed graphene sheets in the cement paste improved the growth of hydration products, which resulted in the establishment of a more compact layer-like microstructure. It is well-known that a synergistic response in a matrix is produced by hybrid fibers due to the combined benefits of individual fibers. The main objective of utilizing hybrid fibers in FRCBMs is to resist cracks at various cracking levels and at various loading phases. It was reported by Lawler et al. [221] that the failure mechanism of macro-FRCBMs can be modified by the micro-fibers due to the reduction in crack width and the production of multiple cracks. The presence of small inter-fiber spacing between micro-fibers might enhance the interactions between cracks and micro-fibers. Xu et al. [11] described that incorporating MWCNTs into cement hydration products could result in the generation of a secondary micro structural interface with a more efficient network due to nucleation and the filling effect. Meanwhile, the stress transfer ability and bridging effect of MWCNTs could efficiently resist nano crack initiation and hence improve initial cracking toughness. Furthermore, the presence of MWCNTs in a matrix could enhance the bonding ability of PVA–steel fibers and cement matrixes, leading to an increased and unstable fracture toughness. Cao et al. [33] studied the mechanism of multiscale hybrid fiber-reinforced CBMs by combining the fracture process with multiscale hybrid fibers that resist cracks. At the crack initiation stage, the micro-cracks were resisted by CWs that enhanced the initial cracking toughness. After that, the combined influence of CWs and PVA–steel fibers restrained the crack expansion and improved the unstable fracture toughness. With a further increase in crack width, enough energy was consumed to result in fiber rupture and fiber pull-out effects.

Researchers have also studied the effect of fibers on the tensile fracture of CBMs [87,238,239,240,241]. Rios et al. [238] analyzed the tensile fracture behavior of FRCBMs using various types (micro and macro) of fibers. It was found that a hybrid reinforced mix (50% micro-fibers and 50% macro-fibers) improved fracture behavior due to the increased cracking strength of the matrix caused by the presence of micro-fibers and the increased deformations before the debonding of the macro-fibers. Kang et al. [87] investigated the influence of fiber content on the tensile fracture of FRCBMs and reported an increasing pattern in the tensile properties of the matrix with the increasing fiber content.

## 8. Conclusions and Future Prospects

### 8.1. Conclusions

In this study, the fiber characteristic and various factors affecting fracture parameters of FRCBMs were described. Many results were collected from various studies and assessed while concentrating on fracture properties with the objective to increase the understanding of the different factors that influence the fracture behavior of FRCBMs. Moreover, the process of fiber effects on crack resistance was described, and recommendations for the future were given. A summary of our conclusions are as follows:

(1) Scientometric analysis revealed that the publications relevant to the current study were mostly in Engineering and Materials Science area. A gradual increase in publications was observed on the fracture properties of CBMs and FRCBMs. However, an abrupt hike was observed in the last decade. From the literature, it was found that the top five most widely used keywords were concrete, reinforced concrete, fracture, fracture mechanics, and fracture energy. It was clear from the visualization network that fibers have a significant connection with the fracture properties of FRCBMs and could comprise the factor with the most influencing on fracture performance.
(2) The incorporation of fibers has a considerable effects on the fracture performance of FRCBMs. Fiber type, tensile strength, morphology, and content can affect fracture properties. Micro-fibers can resist micro-cracks, interfacial modifications, and filling effects in a matrix to enhance fracture properties. Usually, with increasing microfiber content, fracture parameters first increase and then decrease. Hence, to achieve the best fracture parameters, one must find the optimum fiber content.
(3) Compared to micro-fibers, the availability of macro-fibers in a matrix can significantly enhance fracture properties. The best fracture properties can be achieved with steel fibers. Mostly, with an increase of macro-fiber content, fracture parameters improve.
(4) Multiscale hybrid fibers in CBMs have the ability to resist cracks at various scales and hence enhance fracture parameters, particularly initial fracture toughness.
(5) The results of fracture parameters from various studies revealed that different ingredients in CBMs, including the w/c, d_m_, and type of aggregate, can affect the fracture parameters of FRCBMs.
(6) Mostly, increases in the w/c lead to decrease the fracture parameters of FRCBMs while a lower w/c decreases defects in the ITZ and thus enhance the fiber–matrix interfacial bond strength.
(7) The effect of d_m_ is that a large d_m_ usually increases the crack bypass distance or energy consumption of broken aggregate, but a higher d_m_ might alter the dispersion of fibers in a matrix. Generally, it has been found that fibers contribute more to fracture properties in FRCBMs than d_m_.
(8) The influence of aggregate type on fracture parameters depends on the aggregate’s characteristics. For example, a lightweight aggregate reduces fracture energy, while a crumb rubber aggregate improves fracture energy but decreases fracture toughness.
(9) The main crack-resisting mechanisms that occur in FRCBMs are fiber rupture, fiber bridging, fiber pull-out, fiber stress transfer effect, and crack deflection.
(10) Micro-fibers have some additional advantages like a filling effect and ITZ improvement. Porosity can be reduced and pore structures can be enhanced due to the filling effect, while aggregate–cement bond strength can be improved via interfacial modification.
(11) With macro-fibers, besides their inherent properties, their addition to CBMs can enhance fracture performance due to the mechanical interlocking of different fibers.
(12) Fiber hybridization not only provides crack resistance at a single stage like a single fiber but also enhances the fracture performance of FRCBMs with the combined effect of hybrid fibers. Specifically, hybrid fibers containing micro-fibers, in addition to providing crack resistance, can improve fiber–matrix bond strength.

### 8.2. Research Prospects

Currently, the use of fibers of a single type and size is effective at particular levels/scales in CBMs. However, the use of multi-scale hybrid fibers in CBMs is gaining the attention of researchers for achieving multi-level reinforcement effects. Therefore, further studies are still needed in the following areas to promote the use of multi-scale hybrid fiber in CBMs.

1. Durability aspect: The addition of various types of hybrid fibers is effective for fracture properties. However, the physical durability of CBMs with the addition of hybrid fibers must be considered, especially for organic natural fibers like bagasse, hemp, jute, coconut, bamboo, wool, coir, banana, hemp, palm, and sisal. Therefore, it is recommended for further studies to evaluate the fracture characteristic of FRCBMs under freezing and thawing actions, water percolation/permeability, and temperature stresses, i.e., the high heat of hydration. Additionally, the exploration of the fracture properties of FRCBMs is required to evaluate their chemical durability to challenges like alkali-aggregate reactions, sulphate attacks, chloride ingresses, delayed ettringite formation, and the corrosion of reinforcement because FRCBMs are commonly used in these critical environments.
2. Fiber dispersion characteristics: A correlation between fracture properties and fiber dispersion characteristics for FRCBMs needs to be developed in the future because fiber orientation and distribution in the matrix have key functions for the crack growth path.
3. Fracture mode: The type-II fracture (slip-open) and type III fracture (tear) behavior of FRCBMs should be determined because shear failure, in addition to flexural failure, is another fundamental problem of structures.
4. Computer tools: Computing software and tools like the machine-learning approach for the analysis of crack occurrence, crack morphology, and crack propagation path should be applied, as seen in previous studies focusing the purpose on forecasting the fracture behavior of various FRCBM types in engineering and scientific applications.
5. Raw materials for sustainable construction: Focus must be given to the selection of hybrid fibers from raw material like waste steel fibers, waste rubber, recycled plastic, recycled aggregate, steel slag powder, and waste glass powder for the improved fracture performance of sustainable FRCBMs.
6. High temperature performance: At the present stage, the fracture properties of FRCBMs with the addition of multi-scale hybrid fibers under high temperature are still limited. Therefore, it is recommended to study the fire resistance of hybrid fibers in CBMs under fire for structural applications.

## Figures and Tables

**Table 1:** Keyword co-occurrence.

S/N	Keywords	Co-Occurrence	Total Link Strength
1	Fracture	845	5389
2	Concretes	832	5271
3	Fracture mechanics	555	3532
4	Cracks	544	3591
5	Reinforced concrete	489	3371
6	Fracture toughness	441	2908
7	Concrete	391	2468
8	Fracture energy	339	2375
9	Mortar	317	1761
10	Cements	282	1788
11	Reinforcement	271	1884
12	Fibers	270	2203
13	Aggregates	262	1878
14	Brittleness	254	1990
15	Steel fibers	220	1814
16	Fiber-reinforced materials	210	1767
17	Concrete aggregates	202	1364
18	Concrete beams and girders	199	1270
19	Concrete construction	168	954
20	Crack propagation	153	985
21	Fracture property	147	1173
22	High performance concrete	141	1122
23	Microstructure	131	768
24	Concrete testing	128	948
25	Elastic moduli	125	792

**Table 2:** Authors with the highest numbers of citations.

S/N	Author	Documents	Citations	Average Citations	Total Link Strength
1	Wu Z.	26	1398	54	26
2	Shah S.P.	11	1378	125	2
3	Elices M.	10	1251	125	6
4	Planas J.	10	1065	107	8
5	Gálvez J.C.	22	733	33	36
6	Zhang J.	34	671	20	25
7	Li Q.	28	550	20	18
8	Schlangen E.	20	515	26	20
9	Keršner Z.	14	468	33	0
10	Enfedaque A.	17	467	27	32
11	Alberti M.G.	16	443	28	32
12	Karihaloo B.L.	12	428	36	0
13	Chen B.	12	413	34	5
14	Dong W.	12	403	34	19
15	Wang Y.	21	367	17	13
16	Li W.	14	366	26	7
17	Reis J.M.L.	18	362	20	0
18	Yu J.	11	349	32	11
19	Zhou X.	11	347	32	11
20	Hu X.	14	338	24	16

**Table 3:** Articles with most citations.

S/N	Document	Title	Publication Year	Citations	Links	References
1	Chen J.F. (2001)	Anchorage Strength Models for FRP and Steel Plates Bonded to Concrete	2001	851	3	[77]
2	Sim J. (2005)	Characteristics of Basalt Fiber as a Strengthening Material for Concrete Structures	2005	637	2	[84]
3	Yuan H. (2004)	Full-Range Behavior of FRP-to-Concrete Bonded Joints	2004	537	5	[82]
4	Konsta-Gdoutos M.S. (2010a)	Highly Dispersed Carbon Nanotube Reinforced Cement Based Materials	2010	521	2	[10]
5	Wriggers P. (2006)	Mesoscale Models for Concrete: Homogenisation and Damage Behaviour	2006	411	0	[85]
6	Konsta-Gdoutos M.S. (2010b)	Multi-Scale Mechanical and Fracture Characteristics and Early-Age Strain Capacity of High Performance Carbon Nanotube/Cement Nanocomposites	2010	373	2	[78]
7	Dias D.P. (2005)	Fracture Toughness of Geopolymeric Concretes Reinforced with Basalt Fibers	2005	274	2	[86]
8	Wu Z. (2002)	Stress Transfer and Fracture Propagation in Different Kinds of Adhesive Joints	2002	266	3	[79]
9	Kang S.-T. (2010)	Tensile Fracture Properties of an Ultra High Performance Fiber Reinforced Concrete (UHPFRC) with Steel Fiber	2010	176	2	[87]
10	Wu Z. (2016)	Effects of Steel Fiber Content and Shape on Mechanical Properties of Ultra High Performance Concrete	2016	172	2	[80]
11	Ferracuti B. (2007)	Interface Law for FRP-Concrete Delamination	2007	171	4	[88]
12	Banthia N. (2004)	Hybrid Fiber Reinforced Concrete (HyFRC): Fiber Synergy in High Strength Matrices	2004	171	1	[89]
13	Yoo D.-Y. (2013)	Effect of Fiber Content on Mechanical and Fracture Properties of Ultra High Performance Fiber Reinforced Cementitious Composites	2013	155	3	[90]
14	Xu H.H.K. (2001)	Strong and Macroporous calcium phosphate Cement: Effects of Porosity and Fiber Reinforcement on Mechanical Properties	2001	141	0	[91]
15	Kizilkanat A.B. (2015)	Mechanical Properties and Fracture Behavior of Basalt and Glass Fiber Reinforced Concrete: An Experimental Study	2015	134	5	[83]
16	Wu Z. (2003)	Fracturing Behaviors of FRP-Strengthened Concrete Structures	2003	119	1	[81]
17	Capozucca R. (2010)	Experimental FRP/SRP–Historic masonry Delamination	2010	118	4	[92]
18	Kabay N. (2014)	Abrasion Resistance and Fracture Energy of Concretes with Basalt Fiber	2014	117	3	[93]
19	Parveen S. (2015)	Microstructure and Mechanical Properties of Carbon Nanotube Reinforced Cementitious Composites Developed Using a Novel Dispersion Technique	2015	114	2	[94]
20	Zou B. (2015)	Effect of Ultrasonication Energy on Engineering Properties of Carbon Nanotube Reinforced Cement Pastes	2015	111	2	[95]

**Table 4:** Top active research countries based on documents and citations.

S/N	Country	Documents	Citations	Total Link Strength
1	China	681	12,487	83,604
2	United States	417	12,696	68,248
3	Italy	134	3640	22,260
4	United Kingdom	119	4002	24,983
5	Australia	114	2957	29,224
6	India	108	1263	21,838
7	Turkey	99	3031	18,093
8	Iran	95	1380	17,696
9	Spain	91	3125	28,930
10	France	86	2018	14,584
11	Japan	78	2822	6639
12	Germany	76	2240	9233
13	South Korea	68	2203	10,590
14	Hong Kong	57	2651	13,499
15	Czech Republic	56	1189	13,474
16	Brazil	54	950	5631
17	Canada	51	1203	5564
18	Portugal	45	1046	5192
19	Netherlands	42	1354	8601
20	Greece	24	1472	3318

**Table 5:** Fiber properties [102,103,104,105,106,107].

Material Category			Tensile Strength (MPa)	Ultimate Elongation (%)	Elastic Modulus (GPa)	Density (g/cm^3^)
**Metallic**		**Steel**	345–2850	0.5–3.5	200–210	7.65–7.85
Polymers	Synthetic	Polypropylene	240–760	15–18	1.5–10	0.90–0.95
		Polyvinyl alcohol	800–2500	5.7–7.0	29–42	1.2–1.3
		Polyethylene	80–3500	3–100	5–113	0.92–0.97
		Nylon	440–1000	16–20	4.1–5.2	1.13–1.41
		Polyester	580–1100	35.0	15.0	1.22–1.38
		Aramid	2300–3500	2.0–4.5	63–120	1.38–1.47
		Polyethylene terephthalate	420–450	11.2	3.1–10	1.3–1.4
		Acrylic	270–1000		13.8–19.3	1.16–1.18
	Natural	Palm	21–60		0.6	1.3–1.46
		Hemp	270–900	1.0–3.5	23.5–90	1.4–1.5
		Banana	500	1.5–9	12.0	1.4
		Jute	250–350	1.5–1.9	26–32	1.3–1.5
		Coconut	120–200	25.0–10.0	19–26	0.87–1.4
		Abaca	400–980	1.0–10	6.2–20	1.5
		kenaf	223–930	1.5–2.7	14.5–53	1.4
		Sisal	280–750	3.0–5.0	13–26	1.34–1.45
		Bagasse	222–290	1.1	17–27	1.3
		Wool	160		3.5	1.3
		Bamboo	140–800	2.5–3.7	11.0–32	0.6–1.1
		Flax fabric	500–1500		50–70	1.5
		Cotton	390–600	6.0–10	5.8–11	1.5–1.6
		Coir	95–230	15–51.4	2.8–6.0	1.15–1.46
Inorganic		S-glass	4020–4650	5.4	86.9	2.46–2.49
		AR-glass	3240	4.4	73	2.7
		C-glass	3310	4.8	69	2.6
		E-glass	3100–3800	4.8	72.4	2.5–2.62
		Basalt	3000–4840	3.0–3.15	89–110	2.65–2.80
		Boron nitride	2100		345	7.65–7.85
		Alumina	1700–2000	0.4	300–380	3.3–3.95
		Silicon nitride	2500–4800		195–300	
		Asbestos	620		160	2.55
		Silicon carbide	2200–3450		221–250	2.5–2.7
		Alumina-silica	1590–2550	0.8–1.0	200–248	3.4
Carbon fibers		Carbon nanotube	11000–63000		1000–1800	
		Rayon	500–1500	2.5	35–60	1.4–1.7
		Polyacrylonitrile	2500–7000	0.6–2.5	250–500	1.8–1.9
		Mesophase pitch	1500–3500	0.3–0.9	200–900	1.6–2.2
		Graphene	130000		1000	

Note: S-glass: structural glass; AR-glass: alkali-resistant glass; C-glass: chemical glass; E-glass: electrical glass.

**Table 6:** Summary of fibers used for fracture properties of fiber-reinforced CBMs (FRCBMs).

Fiber Type	Matrix	Researcher
Multiwall carbon nanotubes	Cement Mortar	Gdoutos et al. [9]
Multiwall carbon nanotubes and graphene sheets	Cement Paste	Liu et al. [194]
Graphene oxide	Recycled aggregate concrete	Luo et al. [195]
Hooked-end steel fiber	Self-compacting concrete	Ghasemi et al. [196,197]
Hooked-end steel fiber	High-strength concrete	Kazemi et al. [34]
Hooked-end steel fiber	High-strength concrete	Kumar et al. [198]
Steel and polypropylene (PP) fibers	Concrete	Bencardino et al. [199]
Copper-plating steel fibers	Reactive powder concrete	Su et al. [200]
Sheet-wave type steel fiber	Recycled aggregate concrete	Xie et al. [201]
Steel fiber	Recycled aggregate concrete	Guo et al. [202]
Polypropylene fiber	High-strength concrete	Cifuentes et al. [203]
Brucite fiber	Cement Paste	Yang et al. [204]
Basalt and glass fibers	Concrete	Arslan [205]
Basalt and polypropylene hybrid fibers	High-performance concrete	Smarzewski [28]
Multiwall carbon nanotubes and polyvinyl alcohol-steel hybrid fibers	Ultra-high-toughness cementitious composites	Xu et al. [11]
Calcium carbonate whisker and polyvinyl alcohol-steel hybrid fibers	Cement mortar	Cao et al. [33]

## Data Availability

Not applicable.

## Abbreviations

AR-glass	Alkali-resistant glass
CBMs	Cement-based materials
C-glass	Chemical glass
CNFs	Carbon nanofibers
CNTs	Carbon nanotubes
CSV	Comma separated values
CW	Calcium carbonate whisker
d_m_	Maximum aggregate size
E-glass	Electrical glass
FRCBMs	Fiber-reinforced cement-based materials
FRP	Fiber-reinforced polymer
GO	Graphene oxide
HPC	High-performance concrete
HSC	High strength concrete
ITZ	Interfacial transition zone
MWCNTs	Multiwall carbon nanotubes
PAN	Polyacrylonitrile
PC	Plain concrete
PE	Polyethylene
PET	Polyethylene terephthalate
PP	Polypropylene
PVA	Polyethylene terephthalate
SCC	Self-compacting concrete
SFRC	Steel fiber-reinforced concrete
S-glass	Structural glass
SRP	Steel-reinforced polymer
SWCNTs	Single-wall carbon nanotubes
UHPC	Ultra-high-performance concrete
w/c	Water–cement ratio
